# Supplementary material for: Design and validation of a new Healthcare Systems Usability Scale (HSUS) for clinical decision support systems: a mixed-methods approach
Source: BMJ Open. 2023 Jan 30;13(1):e065323. doi: 10.1136/bmjopen-2022-065323 (PMC9887724; doi:10.1136/bmjopen-2022-065323)
Supplement: Author's manuscript [file bmjopen-2022-065323.draft_revisions.pdf]

BMJ Open is committed to open peer review. As part of this commitment we make the peer review history of every article we publish publicly available.

When an article is published we post the peer reviewers' comments and the authors' responses online. We also post the versions of the paper that were used during peer review. These are the versions that the peer review comments apply to.

The versions of the paper that follow are the versions that were submitted during the peer review process. They are not the versions of record or the final published versions. They should not be cited or distributed as the published version of this manuscript.

BMJ Open is an open access journal and the full, final, typeset and author-corrected version of record of the manuscript is available on our site with no access controls, subscription charges or pay-per-view fees (http://bmjopen.bmj.com).

If you have any questions on BMJ Open's open peer review process please email info.bmjopen@bmj.com

BMJ Open

Design and validation of a new Healthcare Systems Usability Scale (HSUS) for Clinical Decision Support Systems: a mixed methods approach

Journal:	*BMJ Open*
Manuscript ID	bmjopen-2022-065323
Article Type:	Original research
Date Submitted by the Author:	13-Jun-2022

Complete List of Authors:	Ghorayeb, Abir; University of Oxford, Nuffield Department of Clinical Neurosciences<br>Darbyshire, Julie; Oxford University, Department of Primary Care Health Sciences<br>Wronikowska, Marta Weronika; University of Oxford, Nuffield Department of Clinical Neurosciences<br>Watkinson, Peter; Oxford University Hospitals NHS Trust, Kadoorie Centre for Critical Care research and Education

Keywords:	Information technology < BIOTECHNOLOGY & BIOINFORMATICS, Health informatics < BIOTECHNOLOGY & BIOINFORMATICS, Health & safety < HEALTH SERVICES ADMINISTRATION & MANAGEMENT, QUALITATIVE RESEARCH, Telemedicine < BIOTECHNOLOGY & BIOINFORMATICS

SCHOLARONE™
Manuscripts

I, the Submitting Author has the right to grant and does grant on behalf of all authors of the Work (as defined in the below author licence), an exclusive licence and/or a non-exclusive licence for contributions from authors who are: i) UK Crown employees; ii) where BMJ has agreed a CC-BY licence shall apply, and/or iii) in accordance with the terms applicable for US Federal Government officers or employees acting as part of their official duties; on a worldwide, perpetual, irrevocable, royalty-free basis to BMJ Publishing Group Ltd ("BMJ") its licensees and where the relevant Journal is co-owned by BMJ to the co-owners of the Journal, to publish the Work in this journal and any other BMJ products and to exploit all rights, as set out in our licence.

The Submitting Author accepts and understands that any supply made under these terms is made by BMJ to the Submitting Author unless you are acting as an employee on behalf of your employer or a postgraduate student of an affiliated institution which is paying any applicable article publishing charge ("APC") for Open Access articles. Where the Submitting Author wishes to make the Work available on an Open Access basis (and intends to pay the relevant APC), the terms of reuse of such Open Access shall be governed by a Creative Commons licence – details of these licences and which Creative Commons licence will apply to this Work are set out in our licence referred to above.

Other than as permitted in any relevant BMJ Author's Self Archiving Policies, I confirm this Work has not been accepted for publication elsewhere, is not being considered for publication elsewhere and does not duplicate material already published. I confirm all authors consent to publication of this Work and authorise the granting of this licence.

For peer review only

Design and validation of a new Healthcare Systems Usability Scale (HSUS) for Clinical Decision Support Systems: a mixed methods approach

Abir Ghorayeb*[1], Julie L. Darbyshire[1], Marta Wronikowska[1], Peter Watkinson[1, 2]

[1] Critical Care Research Group, Nuffield Department of Clinical Neurosciences, University of Oxford, Oxford, UK.

[2] Adult Intensive Care Unit, Oxford University Hospitals NHS Foundation Trust, Oxford, UK.

**Corresponding Author*

Abir Ghorayeb: Abir.Ghorayeb@bristol.ac.uk

Keywords: Usability scale, SUS, CDSS, mixed research methods, questionnaire, clinical context

ABSTRACT (273 words)

Objective: To develop and validate a questionnaire to assess the usability of Clinical Decision Support Systems (CDSS) and to assist in the early identification of usability issues that may impact patient safety and quality of care.

Method: A usability scale was designed and distributed to hospital staff in the United Kingdom. Mixed methods were adopted to develop and validate the questionnaire. The questionnaire was based on evaluations of a CDSS with reference to literature on Health

For peer review only - http://bmjopen.bmj.com/site/about/guidelines.xhtml

Information Systems (HIS) Usability. The qualitative study involved scale item development, content (panel of 7 experts), and face validity (10 participants). Pilot testing (78 participants) established construct validity using factor analysis and facilitated estimates for reliability and internal consistency using the Cronbach's alpha coefficient.

Results: Face and content validity contributed to the development and improvement of scale items. The resulting Healthcare Systems Usability Scale (HSUS) proved quick to complete, easy to understand and was mostly worded by potential users. Exploratory analysis revealed 4 factors related to patient safety: task execution, alerts or recommendations accuracy, the effects of the system on workflow, and ease of system use. These separate into four subscales: patient safety and decision effectiveness (7 items), workflow integration (6 items), work effectiveness (5 items), user control (4 items). These factors affect the quality of care and clinician's ability make informed and timely decisions when using CDSS. The HSUS has a very good reliability with global Cronbach's alpha 0.914 and between 0.702 and 0.926 for the four subscales.

Conclusion: HSUS, a valid and reliable tool for usability testing of CDSS, will be useful for the early identification of usability issues that may cause medical adverse events.

STRENGHTS AND LIMITATIONS OF THIS STUDY

- First valid and reliable usability scale for health electronic systems
- iterative, user-centred approach and mixed research methods
- Designed with potential users
- Four subscales help pinpoint usability issues
- Relatively small number of participants for the pilot study

For peer review only - http://bmjopen.bmj.com/site/about/guidelines.xhtml

INTRODUCTION (7309 words)

Clinical decision support systems (CDSS) are defined as systems that "*provide clinicians or patients with computer-generated clinical knowledge and patient-related information, intelligently filtered or presented at appropriate times, to enhance patient care*"[1]. To benefit from the extensive capabilities of artificial intelligence, health organisations develop CDSS to improve health outcomes for patients and to assist clinicians make appropriate and informed decisions. Ultimately, the intent of any CDSS is to improve clinical efficiency and safety by avoiding adverse events or errors [2]. Clinicians, often working under pressure, are concerned with relevant information access, efficient task completion and better collaboration or communication with colleagues. The design of the CDSS or health information systems (HIS) in general is critical. Poor usability may significantly impact patient care and safety [3-5]. Usable HIS, and more particularly CDSS, would be expected to reduce medical errors, increase efficiency, reduce the cognitive workload of clinicians, and improve patient safety and care. This can be achieved through intuitive design and easily accessible pertinent information that allows users to make timely and informed decisions. Expectations surrounding CDSS development often are too optimistic.

Adoption of CDSS by clinicians is limited due mainly to their poor usability [6, 7], the high volume and complexity of data to analyse, and the failure to fit new systems within existing clinical workflows [6, 8]. Other factors related to system utility, patient safety and impact on professional skills development may also influence the acceptance and use of the CDSS technology [6, 9]. Clinicians need to perceive the benefit of patient safety or improved outcomes to be keen to adopt a new CDSS [9, 10]. They are prepared to learn a hard to use

For peer review only - http://bmjopen.bmj.com/site/about/guidelines.xhtml

or complex system if it is perceived as useful and helpful in complex situations [6]. However, poor usability may impact patient safety, lead to frustrated users, workflow disruptions, increase of medical errors and decreased efficiency [3-5]. Consequently, designers must be mindful of the various and varying needs of clinicians, taking into consideration patient safety and quality of care.

Usability

Usability is defined by the international organisation for standardisation (ISO) as: "*the extent to which a product can be used by specified users to achieve specified goals with effectiveness, efficiency, and satisfaction in a specified context of use*" [11, 12]. It is generally seen as the ability of the technology to allow users to achieve their tasks safely, effectively, efficiently, and pleasantly within a specified context or environment [13, 14].

Different usability aspects appear to be critical for healthcare system acceptability. Patient safety, prevention of medical errors, information exchange, collaboration between colleagues and the complexity of navigation through the system can all influence the clinician cognitive workload. Previous research work on usability of HIS and CDSS suggests that poor usability contributes to the decreased cognitive performance of clinicians [15, 16], low efficiency [17], workflow interruption or disorientation [6, 8, 9, 18, 19], raised medical error risks [20], and higher numbers of adverse events [21]. The electronic health record (EHR), which makes available to clinicians vast amounts of patient data and other information that could be used by CDSS is increasingly suffering from poor usability and safety issues [22, 23]. EHRs are often perceived by users as difficult to use, frustrating, and a source of patient safety hazards, and systems that do not integrate into clinical environments and personal workflow well [24].

For peer review only - http://bmjopen.bmj.com/site/about/guidelines.xhtml

Detecting usability issues in the early stages of CDSS development can reduce or highlight human errors and improve patient safety. It is critical to find an adequate way to involve clinicians in the design process from the beginning. However, usability engineering methods are hard to apply and can be time consuming due to the use of various technology systems, complexity of tasks to evaluate, patient and health professionals' recruitment process. They also need to be conducted within a heterogeneous group of healthcare staff with different backgrounds, needs, skills and experiences [4, 25, 26]. Most of the usability evaluation studies of CDSS to date have used conventional usability methods. Interviews or focus groups [27], non-validated questionnaires [4, 27], or standardised tools like the Systems Usability Scale (SUS) [28] are all commonly used to evaluate CDSS. To the best of our knowledge, specific standards for evaluating the user centred design of CDSS do not exist [29], and different institutions, organisations or vendors use their own guidelines. For example, Russ et al [30] developed a usability testing method to evaluate health technology software influenced by human factors techniques and a few studies have tried to develop non-validated usability questionnaires to assess the usability of HIS [4, 27, 31]. There is therefore a critical need to improve usability standardisation practice for CDSS, and health technology systems in general [22, 32].

Need for a new usability scale for clinical interfaces.

Usability is measured by evaluating the interaction between users, tools, tasks, and the context of use. Various researchers have developed measurements tools for usability aspects such as efficiency, satisfaction, and learnability [33, 34]. Questionnaires are the most often applied usability test due to the simplicity of gathering subjective experiences of a large group of participants in a timely and cost-effective way [35]. However, standardised usability

For peer review only - http://bmjopen.bmj.com/site/about/guidelines.xhtml

questionnaires such as the Questionnaire for User Interface Satisfaction (QUIS) [36] or System Usability Scale (SUS) [28] focus on evaluating the user interface of the system. They do not take into account the wider context of use which is in this case the clinical work [35]. One of the most widely used scale is still the SUS which was originally developed to evaluate the ease of use of systems [28]. The SUS is a standardised questionnaire which consists of ten statements with which participants specify their agreement or disagreement on a Likert scale. Usually, it is used as a primary method of data collection in interface evaluations but sometimes it is one of several methods of data collection.

Due to the lack of a validated tool which takes into consideration the usability aspects related to the clinical context, several studies have used the SUS to assess users' satisfaction of HIS or CDSS [9, 10, 32, 37, 38]. In Nair et al [7] the SUS was applied to evaluate the usability and ease of use of a CDSS for chronic pain management in primary care but identified difficulties in interpretation of the meaning of the score due to the complexity of the clinical task. In the authors' opinion, the SUS neither connects, nor interacts well with the behaviours of the participants observed during the assessment sessions. Another research study [9] used an iterative evaluation of a CDSS through two phases in clinical settings and noticed that the system was rated as usable in the first phase and more usable in the second phase on the SUS while the accompanying qualitative study indicates different degrees of usability in each phase. In phase 1 participants reported the interaction with the system was not intuitive, and that it was time consuming to find the pertinent information. Some of them suffered from a lack of information or additional details needed in the decision-making process. Clinical participants considered the systems disruptive to workflow and felt it took them longer to follow the CDSS' recommendations which in turn limited their use of the CDSS. These results

For peer review only - http://bmjopen.bmj.com/site/about/guidelines.xhtml

were congruent with findings from an earlier phase of the HAVEN project [39] which confirmed that the SUS score may not reflect improvements in interface performance directly. Therefore, the SUS and existent scales are not reflective of the usability in the real world as it does not consider the context of use (in this case, the clinical context). Furthermore, they do not allow evaluators to identify specific usability problems that can be targeted for improvement.

Researchers have previously tried to address this problem by generating their own questionnaire or survey to evaluate the usability of HIS and have used these questionnaires without a clear or systematic validation [4, 27, 31].

A usability scale specifically designed to assess systems in clinical settings can help identify usability problems in an early stage of development or implementation that, if not addressed, may otherwise lead to medical adverse events. To the best of our knowledge, there is no instrument or scale to measure usability of CDSS or HIS that integrates differential usability variance such as patient safety and quality of care. Therefore, there is a critical need for a CDSS/HIS usability scale that takes into consideration different usability aspects, and which is also easy and quick to complete and to analyse. An ideal scale would offer an overview of the usability status of the system with some instructions or information highlighting the specific improvement areas on which to focus.

The aim of this paper is to present the processes used to develop and validate a usability scale that takes into consideration the clinical context.

This scale development work is based on a review of the CDSS and HIS usability literature [40] and on our practical experience from usability testing a CDSS system. This Healthcare Systems

For peer review only - http://bmjopen.bmj.com/site/about/guidelines.xhtml

Usability Scale (HSUS) was designed within the context of the evaluation of a complex CDSS, HAVEN [39, 41]. HAVEN has been designed to be a fully integrated health information system that combines an early warning score with a navigable user interface. Prior to developing the HSUS we evaluated HAVEN for usability, understanding, efficiency, and accuracy. We used the results from these assessments to refine the interface design through an iterative process. Whilst we demonstrated good performance indicators for the HAVEN system, we identified a gap in current evaluation methods. Specifically, we found the common methods used to evaluate usability of information systems lacked specificity in the clinical context. Our aim therefore was to develop a scale suitable to evaluate the usability of any healthcare system.

METHODS

The scale development was iterative. A demographic questionnaire was designed to include a variety of users. To develop a valid, reliable and worthwhile usability scale for HIS in general, and CDSS in particular, mixed research methods were used. The main phases in the development and validation of the HSUS are summarised in Figure 1.

We proceeded as the following:

a) We established the theoretical background based on a literature review and previous usability evaluation sessions of the HAVEN system and authors' experience. As an output of this stage, we developed the first draft of the questionnaire items.

b) We assessed face validity and content validity of the scale using 10 health professionals and a panel of 7 experts in Usability and questionnaire writing for health purpose. These were part of the qualitative research study.

For peer review only

c) We analysed the construct validity of the new scale through pilot testing, factor analysis and internal consistency of the scale. This was the basis for the quantitative research study.

Patient and Public Involvement

Two lay members of the public were part of the HAVEN project management committee and were supportive of the need for a Healthcare Systems Usability Scale.

Content validity

A crucial phase in the validity of a new scale is to ensure that key items have not been missed or omitted [42, 43]. The content validity of a questionnaire is usually attained by 'proof reading' by a panel of experts familiar with the construct being measured. Using the panel of experts to review the scale items in depth improves its quality, understandability and representativeness. Using six to ten experts is recommended to generate a consensus on the construct of interest [44].

We invited a consulting committee of experts to review the questionnaire content validity. Experts were chosen based on their knowledge of the usability construct and their experience working in healthcare. The panel included experts in usability assessment of HIS, plain language, design of health content, questionnaire design for usability and health purposes, and clinical informatics. An introduction letter and content validation form were created to define the aim of the scale and explain to the experts what we needed from them. The background questionnaire, the Healthcare System Usability Scale, an introductory letter and the content validation form were sent via email. On completion, all items were returned electronically and comments from the expert panel were either integrated into the

For peer review only - http://bmjopen.bmj.com/site/about/guidelines.xhtml

questionnaire or captured in a separate document. In this phase, we wanted to understand if the questions captured the usability of the CDSS effectively in the clinical context. The experts were asked to simulate filling out the survey while writing notes and comments, check for common errors (such as double-barrelled questions, double negatives, confusing and leading questions), check questions' clearness and relevance to the main construct, check for any missing aspect of the construct in question, check the question distribution and categorisation, and propose any new aspects not covered by the questionnaire. Finally, they were asked to give each question a score from 1 to 10 which refers to the extent each item relates to the usability of the system.

Face Validity

Face Validity refers to the degree to which the purpose of the scale is obvious to the target population or users and seems to be a valid measure for its purpose [45, 46]. Face validity evaluates the questionnaire in terms of "feasibility, readability, consistency of style and formatting and the clarity of the language used" [47, 48]. Ideally, the participants who assess questionnaires for face validity will be representative of the target population (the end users).

The participants representing future end users who were approached to assess face validity were recruited from two hospitals within the Oxford University Hospitals NHS Foundation Trust (OUHFT), the John Radcliffe Hospital and the Churchill Hospital. We recruited through in-person and email invitation. To be eligible we needed participants to be: (1) Health professionals (physician, nurse, physiotherapist, pharmacist or scientist), 2) Have more than three months experience using the local hospital electronic patient record system (SEND or CERNER Millenium®), (3) Willing to participate in the study.

For peer review only - http://bmjopen.bmj.com/site/about/guidelines.xhtml

Prior to the start of the study, all participants signed written informed consent. The question items were presented to allow discussion of the usability and healthcare dimensions to be evaluated or to be included in the scale; ensure the inclusion of any usability missing aspect; review the set of questions and contribute to the usability, understandability and clarity of the question items.

For this project an assessment form was developed to help the interviewer and participants assess questions in terms of clarity, understandability and meaningfulness, layout and style, and the ability of the participants to answer the questions. Face validity of the HSUS was determined by a review of the items iteratively by 10 participants. Suggestions from each participant were incorporated in the next iteration evaluated.

Each participant's interview was recorded for analysis. After each interview, the participant's feedback was analysed and the questionnaire was amended to be evaluated by the following participants (by adding, deleting or amending a question item). The original questionnaire consisted of 28 multiple choice questions and three open ended questions. This was tested iteratively with ten health professionals until data saturation was reached

During the interviews we used the think aloud technique [49]. This requires the participant to explain out loud each thought they have while reading the questions and discussing the different suggested statements. We were looking to learn how participants speak intuitively about system usability, aspects of patient care, and how they interpret statements using their own words. Participants were asked to select the statement they thought best reflected the construct in question, and to change wording, delete statements or add new aspects they thought relevant (Figure 2)**Error! Reference source not found.**.

For peer review only

Pilot testing

The last validation step is to pilot test the scale where participants of the target population complete the questionnaire independently. For the validation and the comparator usability testing of the scale, we used two well-known health systems used at the Oxford University Hospitals NHS Foundation Trust (OUHFT): SEND and CERNER Millenium®. SEND is a CDSS used by health professionals at the hospital to record patient vital signs observations. This is a fully integrated system developed locally. It allows healthcare staff to record and monitor patient vital signs electronically from the bedside. The system integrates with the Trust electronic patient record system and incorporates a simple data entry interface with a visual trend of historic values to aid clinical decision making in hospital. The SEND system also includes limited advice to support escalation processes in the event that physiological deterioration is identified. We also asked participants to assess CERNER Millenium® which is a different HIS also in use at the OUHFT. We know from previous evaluations that healthcare staff rate SEND significantly more usable than CERNER Millenium®. Other work has recognised significant limitations in the visual appearance of the CERNER Millenium® interface which is likely to hinder acceptability and satisfaction in comparison to the simplicity of the SEND interface [50].

In the piloted questionnaire, we added the option of "statement not clear" to identify any statement that might need improvement. The option "Not applicable" was also added as not all HIS or CDSS offer the same functionalities. For example, CERNER Millenium® can be used to record and monitor patients' vital signs observations but does not include a visual graphical

For peer review only - http://bmjopen.bmj.com/site/about/guidelines.xhtml

display to aid interpretation of data. CERNER Millenium® also includes direct links to the patient admissions and treatment records which SEND does not.

Participants were recruited from two hospitals within the Oxford University Hospitals NHS Foundation Trust (OUHFT), the John Radcliffe Hospital and the Churchill Hospital through in-person and email contacts. The participants represented a range of experience. Eligibility criteria were similar to those recruited to assess the HSUS for face validity and consisted of: (1) Health professionals (physician, nurse, physiotherapist, pharmacist or scientist), 2) Have more than 3 months experience using SEND or CERNER Millenium®, (3) Willing to participate in the study. At the beginning of the questionnaire, participants completed and approved a consent form and answered the demographic questions.

The questionnaire was available online and offered as a paper copy. The researchers systematically observed and recorded any difficulties encountered during the process. Data were reviewed to highlight any items that were difficult to understand or generate confusion and to assess the reliability and correlations of the scale. Exploratory factor analyses, which are more convenient for new scales' development [51], were conducted to identify the underlying constructs or factors. Items measuring the same construct were clustered into the same subscale. Finally, the internal consistency and correlation of the whole scale, and for each subscale, was investigated using Cronbach's Alpha coefficient to ensure scale items were consistent.

RESULTS

The first draft of the questionnaire was based on the usability definitions from CDSS literature to cover the different usability aspects including learnability, efficiency, effectiveness,

For peer review only - http://bmjopen.bmj.com/site/about/guidelines.xhtml

usefulness, accessibility and user satisfaction [52, 53]. We also wanted to acknowledge the clinical context in which this system is intended to be deployed so included additional patient safety and quality of care aspects. The main challenge to creating an effective scale involves writing questions which are unambiguous and understandable using the vocabulary of the target population. This first draft was developed to cover the main usability issues which mainly linked to:

- Understanding how the recommendations, scores or alerts are made [18]: Clinicians emphasise the need to comprehend how the CDSS' decisions, scores or alerts were elaborated or developed. It is part of their training to probe for reasons behind results as interpretations of the outcome or critical issues may differ despite apparently identical data. The 'black box' approach is disliked almost universally.

- Ease of access to additional information: The main aim of all CDSS is to provide an intuitive design to allow pertinent information to be easily accessible to facilitate timely informed decisions. Clinicians must be able to locate the specific information they require promptly.

- Perceived utility: one of the main adoption barriers of CDSS is a lack of perception of its utility. Health professionals must feel the new technology will help them improve patient outcomes, improve their decision effectiveness and ensure patient safety.

- Cognitive workload: A CDSS that requires significant mental effort to use is most likely to be rejected by health professionals working in a busy environment.

- Workflow integration: the new system must fit into the workflow of the health professionals and integrate well with current systems and processes.

For peer review only

For peer review only - http://bmjopen.bmj.com/site/about/guidelines.xhtml

> Recommendations, alerts or scores should be provided and displayed in a way that supports the clinical work; otherwise, the system is likely to be seen as a waste of time.

To elaborate the first draft of the questionnaire, we collaborated with five scientists working in the medical field to ensure the inclusion of all construct aspects and the appropriateness of each question.

Content Validity

The first set of 21 discreet choice questions and three open ended questions was reviewed by the panel of experts. All experts emphasized the need for a new, more clinically focused usability scale to assess CDSS and HIS, as evidenced by the following quotations:

> Expert A: *"I don't know of any survey instrument that focuses specifically Electronic decision support systems. Therefore, developing well-researched questionnaires that can help you distinguish between more usable and less usable systems is a worthwhile".*

> Expert B: *"I think the questionnaire can be an excellent contribution to the field. I really like the idea of embedding context as that is critical to perceptions about a system."*

> Expert C: *"I think each of the subscales is relevant to the concept of usability." I also think the respondent burden (the time and effort for respondents to fill out the questionnaire) is fine".*

They commented on the questions' clearness, appropriateness and understandability, and looked for leading, confusing or double-barrelled questions and suggested corrections. The panel of experts were asked to rate each item on a scale of 1 to 10, indicating how closely

For peer review only - http://bmjopen.bmj.com/site/about/guidelines.xhtml

each item related to the system's usability. We were looking to check whether these items or factors need to be weighted or balanced in some manner. Most experts disagreed with the concept of assigning a score, arguing that it relies on the system being evaluated, its functioning, and role. It is up to the researcher or investigator to decide whether certain needs, such as patient safety and decision effectiveness, should be prioritized. Some items are more important than others and data analysis should take this into account. While the HSUS does not weight items, it does allow for the quick and easy identification of the source of usability issues by investigating each subscale separately.

The comments from the expert panel guided us to rethink and reword several of the items. For example, for the first item "I feel satisfied while using the system", some of the experts suggested to interview potential users with the following: "This question makes me feel ……." and ask users to suggest an adjective or to choose from a range of adjectives using the Microsoft Product Reaction Cards [54]. We were also using the term 'complexity of tasks' and the experts wondered how respondents would interpret this. What tasks do they think this system is helping them to manage? Or is the complexity coming from something that's nothing to do with the system? The participants in the face validity phase proposed we exclude the term "complexity of tasks" and replace this with a daily workload assessment. Other advice included separating legibility, findability and understandability as these are distinct needs.

More generally, the panel of experts guided us through our questionnaire development by suggesting corrections, re-ordering of questions, and advising we needed to be more specific and ask only what is necessary for analysis. They suggested additional questions which might be useful for analysis such experience with electronic healthcare systems other than the

For peer review only - http://bmjopen.bmj.com/site/about/guidelines.xhtml

specific system being evaluated. They found the open-ended items easy to understand, and thought they would be useful to detect aspects of the construct not otherwise well represented or omitted elsewhere. They also suggested we evaluate the background questionnaire with potential users and ask them for suggestions.

Three of the seven experts offered further review after usability testing of the amended version. This usability testing altered both the content and structure of the questionnaire. Subsequently a revised version of the questionnaire was drafted, and a usability and understandability test was developed during the Face Validity phase. This amended version of the questionnaire was given to the three experts for a second review and a tele-conference was organised to discuss feedback and agree consensus. We made some additional modifications before the pilot testing phase.

Face Validity

The recruited cohort for this phase represented a range of experience, age and skills. The sample consisted of four nurses with different level of expertise, two physiotherapists, two doctors in training, and two consultants. The final scale consists of 22 items rated on a 7-point Likert scale from 'strongly disagree' to 'strongly agree' including 'not applicable' and 'statement not clear'. Data saturation occurred when participants had no more concerns or new suggestions, and we noticed agreement within the last four participants.

We present direct comments and some examples of participants in Figure 2. For example, in question 5, participants were asked to define 'quality of care' to make sure that we share the same meaning. We were asking the participants a series of questions designed to elicit more information about how framing a question in different ways might affect participants'

For peer review only - http://bmjopen.bmj.com/site/about/guidelines.xhtml

thoughts, understanding and reasons of selection. Each time a person suggested a new item, new terminology, or a statement's correction, the document was changed and reviewed by the next participant. This phase of testing helped us revise some double-barrelled and unclear statements while taking into consideration participants' insights, experience and points of view.

Participants' responses were summarised on a spreadsheet (Table 1) for more detailed analysis. The last four participants selected the same statement and shared the same understanding. In Table 1 we summarise examples of three statements, selected statements, and justification for the selection.

Table 1 Selected examples of items development during Face validity

User number	Question 2	Question 5	Question 9
01 (senior nurse)	I like using SEND	This system is improving the quality of care	I understand how the system derived its recommendations, scores or alerts
02 (Physioterapist)	I like using SEND because of its ease of use / because it is stress free	I am able to provide better quality of care for patients than I could before I had this system	I understand how the system derived its recommendations, scores or alerts
03 (pharmacist)	I like using SEND	This system is improving the quality of care	I understand how the system derived its recommendations, scores or alerts
04 (training doctor)	SEND does not make me feel stressed	I feel that I am able to provide better quality of care for patients than I could before I had this system	The system's recommendations are congruent with clinical current practices and standards/ conform
05 (consultant)	SEND does not increase the barriers to do my work	I feel that I am able to provide better quality of care for patients than I could before I had this system	The system's recommendations are consistent with clinical practices and standards
06 (senior nurse)	SEND does not make me feel stressed	My colleagues and I are able to provide better quality of care for patients than we could before we had this system	The system's recommendations are consistent with clinical practices and standards
07 (training doctor)	SEND is user-friendly	I am able to provide better quality of care for patients by using this system	I understand how the system derived its recommendations, scores or alerts and The system's recommendations are consistent with clinical practices and standards (clinicians should have been using the system for a while to be able to decide this item)

For peer review only - http://bmjopen.bmj.com/site/about/guidelines.xhtml

08 (Doctor)	SEND is user-friendly	I am able to provide better quality of care for patients by using this system	I understand how the system derived its recommendations, scores or alerts and The system's recommendations are consistent with clinical practices and standards
09 (Scientist)	SEND is user-friendly	This system is improving the quality of care	I understand how the system derived its recommendations, scores or alerts and The system's recommendations are consistent with clinical practices and standards
10 (Nurse band 5or 6)	SEND is user-friendly	I am able to provide better quality of care for patients by using this system	I understand how the system derived its recommendations, scores or alerts and The system's recommendations are consistent with clinical practices and standards
**Selected statement**	**SEND is user-friendly**	**I am able to provide better quality of care for patients by using this system**	**Based on the participants' suggestions. This item is splitted in two: I understand how the system derived its recommendations, scores or alerts and**<br><br>**The system's recommendations are consistent with clinical practices and standards**
**Justification**	**"I like using SEND": participants can like the system for many reasons, confusing statement. Participants were hesitating between the two statements: "SEND is easy to use" or "SEND is user friendly". As last statement was suggested by the participants, so we decided to adopt it.**	**"This system is improving the quality of care" is too broad. Participants can't really know if the system is improving the quality of care, but they can judge their proper work. We think there is no real need to add "I feel" at the beginning of the statement as suggested by two participants.**	**Pharmacists and physiotherapists were not interested to know about how the scores are derived but they don't mind knowing. Clinicians, by culture, will never use a system if they don't understand a minimum of how the score and recommendations are derived (they said it clearly). Last few participants suggested to keep the original statement and to add the suggested statement as an independent item.**

The last version of the amended document was discussed with the three experts who had offered to follow the progress of the work. They advised to proceed with the pilot testing and adopt the suggestions, even though some of these suggestions contradicted the panel of experts' advice. For example, in question 14, many experts suggested to avoid the use of the word "*navigate*" as it is classified as computer jargon and suggested the following statement instead: "*It is easy to get to what I need in this system*". However, during the usability sessions users advised the use of the word "*navigate*" which might be justified by the daily use of computer technology by all the participants and health professionals in general. As a result of

For peer review only - http://bmjopen.bmj.com/site/about/guidelines.xhtml

the second round of interviews with the panel of experts, a final draft questionnaire was ready for the pilot testing.

According to the results of the qualitative studies, most of the items were reworded, four items were deleted, and six items were added. The final version of the questionnaire put forward for pilot testing consisted of 23 items.

Pilot testing

The sample consisted of 78 participants aged 18-65 years. Most of the participants use their local electronic healthcare system daily for at least 1-3 hours, have experience in healthcare for more than one year, and work in various healthcare departments as shown in Figure 3.

To investigate if the new HSUS was able to differentiate between a system that is perceived to be hard-to-use (CERNER Millenium®) and a system perceived to be easy-to-use (SEND), we piloted the questionnaire with 37 healthcare professionals using CERNER Millenium® and 41 using SEND. Although the aim was not to evaluate SEND and CERNER Millenium® specifically, the results of the survey revealed that the usability score for CERNER Millenium® was 64 per cent while the general HSUS score for SEND was 81 per cent, with 86 per cent for the workflow integration subscale.

The cumulative distribution function of the subscale "patient safety and decision effectiveness" for SEND system (Figure 4) is an example of the further investigation offered by the HSUS, and it clearly shows a high usability score, with only a few participants scoring less than 80%.

For peer review only

For peer review only - http://bmjopen.bmj.com/site/about/guidelines.xhtml

The HSUS included three optional open-ended questions: "*I would add these functions or features*", "*I would change the following*", "*Please use the space below to provide any other comments*". Participants' comments were concise, informative, to the point, and represent the users' perspectives, as demonstrated in Table 2 collected during the pilot testing phase. The differing usability scores for SEND and CERNER Millenium® can be understood in context from these user comments.

Table 2 Utility of HSUS open-ended questions: examples from SEND vs CERNER Millenium® cohort

CERNER	SEND
**I needed help with**	
-Navigation, layout, more clinically designed (congruent with systems I am used to)<br>-Is not user friendly-too complex, too many different places to go. For example: tasks/interactive view/care plans- all need to be done every shift for every patient but all in different places and you need to search for them in a big list.<br>-Uploading photo's correctly. navigating through the system-finding what relevant to my practice and how to successfully use. it can freeze or be slow. we often miss patients not referred. more information needed upon occasion<br>-Scheduling appointments find notes find coding	-Initial login etc<br>-Nothing<br>-Nothing<br>-Nothing<br>-Nothing<br>-Nothing<br>-Nothing I am happy with using SEND<br>-Nothing currently<br>-Nothing<br>-Correcting data if error used<br>-Nothing
**I would add these functions or features**	
-CERNER should have the "continuous doc" feature added at OUH to make searching much easier<br>-Per shift: all the care plan + tasks + interactive view in the same place and then other things could be added as needed.<br>-instead of having different encounters on the same patient I would have the patient file divided by specialities. Add research function (specialities)	-Total flow whilst on nasal high flow systems<br>-More detailed information regarding normal ranges for individuals. Some 'standard parameters do not apply to all individuals'<br>-Link to labs<br>-Flow rate and FiO2 for high flow nasal oxygen<br>-Accurate recording of patients on HFNC oxygen<br>-Target SpO2 and adjust the traffic light system to reflect this (chronic Respiratory conditions vary so these need to be able to be adjusted.<br>-Flow rate with Airview<br>-Cap refill, Urine output - if applicable<br>-Oxygen therapy should add to the trigger score. Some patients can have a T&T of 0 but be on 8L of oxygen therapy.<br>-If patient has specific O2 ie. COPD aim SATS, to be reflective on SEND screen. Ability to cancel incorrect data after recorded.<br>-When recording blood pressure, there is an option for lying and standing, maybe add the option of sitting up right.<br>-Urine output and fluid balance all under one system to see trends along with the vital signs this may increase compliance.
**I would change the following**	
-Layout, accessibility, observation recording, search function, documentation.<br>-Add on the available functionality such as continuous doc<br>-Not have different encounters -Add a search facility by using coding structure.<br>-Able to build theatre lists and upload Timms data + Bluespier-Add oncology data including chimo radiotherapy	-Better graphic representation<br>-Add in flow rate for high flow oxygen<br>-Increase the login timeout<br>-Make it more like HAVEN<br>-Consideration of link with ICCA for translation of date from one system to the other without duplication. Or the ability to go back further than 4 hrs to input data as it is duplication of records.<br>-Change to NEWs2 regarding oxygen saturations
**Please use the space below to provide any other comments:**	
-It is a poor, pretty unusable system.<br>-more training/update sessions to keep up with the changes. Dr Logon can be temperamental! it slows work efficiently<br>-Generally CERNER has been a fantastic tool to help me to complete my job role. I can quickly check the status of patients and	-Very useful<br>-Nothing to add<br>-I like how if you have starred your patients & discharged them they reappear on your page if readmitted to hospital.

For peer review only - http://bmjopen.bmj.com/site/about/guidelines.xhtml

prioritise them accordingly. In order to ensure more staff use the system for patient referrals and communication, it needs to be much more user friendly/replicate the "look/feel" of more established systems. This will ensure patient data is shared safely and securely<br>-When review of medications is requested there is a field to highlight not medically responsible. This generates lots of lists on power note until reviewed by medic but think this should be placed in a different format rather than producing reams of lists<br>-It feels like a very clunky system which is slow and takes a long while to achieve what is required<br>-It'is really time consumming, slow, not always making our time efficient seems to be all over the place, needs more structure to the components of nursing care that are needed<br>-The safety messages that pop up when you log in have the "Don't show until new info posted" box but this doesn't work – I keep getting the same message for days or weeks despite using it.	-I feel SEND stsem takes away the clinical thinking and the judgement of staff to make clinical decisions<br>-It takes a bit while to learn and remember about various functions on EPR. Also I find it a bit difficult to correct errors of documentation

Factor Analysis

Factor analysis is a statistical method that clusters items into underlying constructs [55]. Each construct is a list of questions or items that can be associated with each other. Authors have argued on exactly how big samples should be as there is no simple rule-of-thumb [56] and it is rarely justified in the literature [57]. Inappropriate sample size might lead to inaccurate findings during the development and validation of a new scale, in particular the identification of the structure of the new scale and its subdivision in latent constructs. The sample can be determined "by the nature of the data, i.e. the stronger the data, the smaller the sample size can be. Strong data display uniformly high communalities without cross-loadings" [58].

To check the sample size's adequacy to undertake exploratory factor analysis for this study, we performed Bartlett's test and the Kaiser-Meyer-Olkin (KMO) measure analyses. The KMO value varies between 0 and 1, with values closer to 0 indicating that factor analysis is inadequate. Exploratory factor analyses, on the other hand, have a high adequacy and can create distinct and reliable subscales or factors when the value is close to one [59]. KMO values between 0.5 and 07 are considered mediocre by Kaiser; 0.7 and 0.8 as good, 0.8 and 0.9 as great, and > 0.9 as superb.

For peer review only

To explore the factor structure of our new HSUS, we used the Principal Component Analysis (PCA) with a Varimax rotation as the extraction method due to its high reliability and lack of error [55]. The inclusion or exclusion of an item in a construct was determined by factor loadings >0.5 [60], eigenvalues>1, the scree plot and the total variance are explained by the domain or factor.

All 23 items of the HSUS were analysed using exploratory factor analysis. The resulting KMO was 0.818, indicating "great" on the Kaiser scale. The Bartlett test was similarly positive with a p-value <0.001 (Figure 5). As the KMO of the HSUS was above 0.8, and all the variables had loadings >0.5, the sample size is considered adequate for Factor Analysis and can be tested to see whether the scale performs as intended.

When Kaiser's criterion and PCA were applied to the HSUS, five factors had eigenvalues ≥ 1.00. The exploratory factors analysis identified five domains or subconcepts which explained 68.62 % of the data variance. Commonalities of the 23 items ranged from 0.515 to 0.867. Four of the factors had at least three items while the fifth only had one. For a better interpretation we built the scree plot which indicated there were 3 to 5 factors which account for the variance (Figure 6).

Only three statements were judged unclear once by two different participants (*"It is easier to make efficient decisions by using (name of the system); (Name of the system)'s recommendations are consistent with clinical practices and standards; (Name of the system) helps improve patient outcomes".* Factor 5 ("*A recommendation (false alert or score) is unlikely to impact patient safety*") was judged twice as an unclear statement and as this item was covered by the rest of the scale, we decided to delete it to re-assess the statistical analysis. This revised analysis shows the best statistically and conceptually appropriate

For peer review only - http://bmjopen.bmj.com/site/about/guidelines.xhtml

solution for the new HSUS is a four-factor scale. The KMO of the final 22 item HSUS is 0.823 (Figure 7).

When Kaiser's criterion and PCA were applied to the 22 items, four factors had eigenvalues ≥1.00 and the EFA results identified 4 factors or sub-concepts where the commonalities of the 22 items ranged from 0.501 to 0.874. This helps to identify and localise the usability issues clearly and easily in each factor.

The first factor evaluates the utility perception of the system. For CDSS this relates to patient safety and decision effectiveness. This factor is formed of seven items, reflecting its importance in system adoption. Factor two includes six items concerned with the ease of use of the system which facilitates integration within the clinical workflow. Factor three with five items reflects the work effectiveness and cognitive workload and the final factor with four items relate to the user's perception of control. The final scale with the factor loadings of the final PCA are shown in Table 3.

For peer review only

For peer review only - http://bmjopen.bmj.com/site/about/guidelines.xhtml

Table 3 Final HSUS with factor loadings and Cronbach's Alpha

Items	Utility: Patient safety and decision effectiveness Factor 1	Ease of Use: Workflow Integration Factor 2	Work Effectiveness Factor 3	User Control Factor4
(System name) helps me work more efficiently	0.734			
(System name) makes it easier to collaborate with colleagues	0.758			
I am able to provide better quality of care for patients by using (system name)	0.613			
It is easier to make efficient decisions by using (system name)	0.706			
(System name) helps improve patient outcomes	0.642			
(System name) helps prevent clinical errors	0.677			
(System name) generates a useful summary view of the patient's current health status	0.503			
(System name) fits well with the way I currently work		0.609		
I found the information provided on the screen understandable		0.818		
I found it easy to navigate through (system name)		0.847		
I can easily remember how to use (system name)		0.801		
The screen layout makes it easy to see each piece of information		0.84		
On the screen, I can find specific information I need quickly		0.803		
(System name) helps me prioritise my daily workload			0.675	
I understand how (system name) creates its recommendations (scores or alerts)			0.67	
(System name) 's recommendations (scores or alerts) are consistent with clinical practices and standards			0.704	
(System name) includes all the relevant information I need			0.678	
I believe the recommendations (scores or alerts) are reliable			0.758	
(System name) supports my decision making rather than dictating it				0.516
It is easy to correct a data entry error in (system name)				0.758
(System name) highlights potential data entry errors				0.781
Recommendations (or alerts) do not unnecessarily interrupt my workflow				0.506
**Cronbah's alpha coefficient**	**0.873**	**0.926**	**0.777**	**0.702**

For peer review only

Internal Consistency and Reliability

To conduct the reliability analysis, we calculated the Cronbach's alpha coefficient which is a measure of the internal consistency of the question scores. It depends on the inter-question correlations and the total number of questions.

Reliability contributes largely to the validity of a questionnaire as it refers to the extent to which the results obtained by the instrument can be reproduced. Internal consistency concerns the degree to which items on the scale or sub-scale are measuring same concept. Reliability can be established using a pilot test with 20 to 30 participants [61]. To study the inter-item correlations within the HSUS we calculated the Cronbach's alpha coefficient[62] which is a measure of the internal consistency of the item scores. Cronbach's alpha was calculated for each subscale as well as for the complete scale. Cronbach's alpha was 0.914 for the HSUS scale, indicating a high inter-items correlation, suggesting the scale is consistently reliable. The Cronbach's alpha of the four subscales also exceeded 0.7, which is the minimum suggested for a new instrument [48, 63] (see Table 3**Error! Reference source not found.**).

DISCUSSION

The HSUS was developed using a rigorous, iterative, and user-centred approach that consisted of face validity, content validity, exploratory factor analysis, and reliability testing. First, we proposed items based upon our experience from previous evaluation of the HAVEN system. We complimented this knowledge with a literature review and discussions with various clinicians working with the research team. A panel of seven usability experts in forms and surveys, plain language, user experience, and health content designers contributed to the content validity. The number of experts for content validity was sufficient to agree consensus

For peer review only - http://bmjopen.bmj.com/site/about/guidelines.xhtml

on the main concept [44]. Three of these experts recommended another review of the revised version at the end of the HSUS usability testing or face validity phase.

Face validity of the HSUS was established by an iterative review of the questions by ten health professionals recruited for a semi-structured interview. Each participant's suggestions were incorporated into the following iteration for evaluation, resulting in a total of five iterations of the scale. Face validity was established when we noticed agreement among the last four participants. The participant variability and concept saturation determined the sample size for the qualitative work [64, 65].

During the content and face validity phases of the HSUS, items were deleted, changed, and new items were suggested to ensure that all system usability aspects were addressed, and the questionnaire items were well designed and written, with no common errors such as double negative, confusing, or double-barrelled items. The number of items in the HSUS ranged from 21 at the outset to 28 for face validity and finally, after statistical evaluation, to 22. The exploratory factor analysis defined 4 factors: patient safety and decision effectiveness, workflow integration, work effectiveness, user control. These factors covered issues related to the patient safety, the ease of use of the system, task's complexity, the alerts or recommendations utility, the effects of the system on the workflow, the quality of care and ability of the clinician to take an informed and timely decision. The HSUS presented a very good reliability evaluation with a global Cronbach's alpha of 0.914 and subscale alphas ranging from 0.702 and 0.926.

Whilst the open-ended questions were optional, they had several advantages when used within the HSUS questionnaire. They allowed participants to independently and creatively comment using their own lexicon without being influenced by the interviewer and it revealed

For peer review only - http://bmjopen.bmj.com/site/about/guidelines.xhtml

important issues to investigate further. Free text responses can also provide quick feedback that may reveal unexpected findings.

Challenges in developing a usability scale in the clinical context are significant, and few or no work or guidelines exist in the literature. The specificity of the target user is the first challenge to face as health professionals are experiencing growing level of stress and cognitive workload. While new technologies may improve the quality of care and assist health professionals in their decision-making, healthcare professionals will continue to reject any technology that they do not perceive its utility or that does not fit appropriately in their workflow [6, 29]. Another challenge in the healthcare environment is patient safety and quality of care, both of which are aspects associated with usability of health information and clinical decision-making systems. When a new healthcare system is established, it should improve patient outcomes while also ensuring the avoidance of errors and adverse effects on patients and/or the wider healthcare system itself. Another challenge is deciding whether these needs must be balanced or whether the necessity of effectiveness (safety, workflow) should be emphasised. This healthcare systems usability scale (HSUS) was specifically created with the intent of taking the context of use into account.

The review of existing CDSS systems, usability scales, and previous research studies urged the need for a short, easy to use, reliable and accurate usability scale for health information systems. To the best of our knowledge, this HSUS is the first validated scale for testing the usability of clinical decision support systems (CDSS) or healthcare information systems (HIS) that also takes into account the specific clinical context. We have shown that the HSUS is an efficient method for collecting subjective opinions and experiences from a large number of potential users, as well as analysing usability issues. Dividing the HSUS into four sub-scales is

For peer review only - http://bmjopen.bmj.com/site/about/guidelines.xhtml

a novel feature that allows evaluators to identify specific usability issues and investigate each subscale separately. The fact that is easy to fill, brief, and can be delivered electronically makes it simpler to reach large numbers of users. The HSUS was created for health professionals with varying degrees of computer ability or literacy, while keeping in mind that they may be busy, distracted, or interrupted.

This scale can provide critical information to address the most important usability challenges to the design and subsequent use of complex clinical decision support systems. In addition to the HSUS, we recommend that other usability testing methods, such as interviews and focus groups, are also required for a more thorough and realistic evaluation, as well as to evaluate whether the participants' behaviours match well with the results from the HSUS. The HSUS will detect early usability issues, identifying the source of problems, and pinpointing the threat rate. It is difficult to assess a system after the initial implementation. Usability scales that are used after a quick review of a new system during a usability test session tend to be less realistic than tools used after a few days or a short-term trial. It may be difficult and inaccurate for users to evaluate systems after their first interaction. It is preferable to use the usability scale a few days after implementation to allow some degree of familiarity to form. This valuable and novel work was developed and validated through several phases

Using the HSUS; customization for real world implementation

The online survey was designed to take 10 to 15 minutes to complete due to the time constraints of the health professionals. Some items may not be applicable to any CDSS or HIS. These items can be omitted. The options “unclear statement” and “not applicable” were added for validation purposes. We needed to know if any statements in the HSUS were

For peer review only - http://bmjopen.bmj.com/site/about/guidelines.xhtml

unclear. For future use of the HSUS, these two options may be omitted, and the final scale will consist of 22 items rated on 5-point Likert scales.

We recommend tailoring the questionnaire to each application and naming the system within the question (replace the word “system”). Depending on the system's functions, the questionnaire should be customized to use the terms "recommendations," "scores," or "alerts." (**Error! Reference source not found.**3)

The complementary information gathered from the background questionnaire could be useful for HSUS data analysis. The background questionnaire should be kept short and include only those questions that are directly relevant to the needs of the study.

The HSUS can be used for the assessment of the severity of usability violation of each subscale. Each item is rated on a 1 to 5 scale; 1= Strongly disagree (crucial usability concern), 2= disagree (major usability concern), 3= neutral (minor usability concern), 4= agree (usability can be improved), 5= strongly agree (no usability concerns). A higher score reflects a higher level of agreement on each item. We calculated the overall group average for each item followed by the average for each subscale. The higher the average score for each subscale, the higher the level of usability perceived by participants. The overall score of this scale is the average of the four subscale scores. A usability score between 20% and 50% indicates a critical need to address the system's usability issues; between 50% and 70% indicates a need to address the system's usability concerns, some of which may be major; between 70% and 90% indicates a good usability score with the potential to improve; and between 90% and 100% indicates an excellent and easy to use system. The researcher or investigator must decide if particular requirements, such as patient safety and decision effectiveness, should be prioritized.

For peer review only - http://bmjopen.bmj.com/site/about/guidelines.xhtml

Decision effectiveness and patient safety were combined statistically and logically due to the relationship of efficiency to safety [29, 66]. If health professional performance is affected, work efficiency is slowed, and quality of care and patient safety may be jeopardised. A lack of usability in this subscale should raise alarms as the use of the system under evaluation might endanger patient safety. This part reflects the perceived utility by the health professionals.

LIMITATIONS

A possible limitation is the relatively small number of participants even though some experts recommend a sample size of 12 to 50 for a pilot study [67-69]. While the KMO-test value revealed that the sample size was sufficient for factor analysis, the findings of the factor analyses may differ with greater number of participants. To strengthen the consistency of the scale and support its standardisation, further work could be undertaken in a larger sample.

This study was limited to healthcare professionals based in the secondary care environment. When used under different contexts or working circumstances, validity testing may be required, but the HSUS could be applied to other types of clinical system in other healthcare settings.

CONCLUSION

In this paper, we have presented novel work to develop a validated and reliable instrument to measure the usability of electronic systems in the healthcare environment. The HSUS can be useful in research institutes and hospitals to improve the usability of a newly designed or implemented CDSS or HIS in an efficient way. Because it has been designed for self-administration and brevity, this scale offers an easy way to assess the usability of clinical systems that can be used alongside other techniques. This offers a better understanding of

For peer review only - http://bmjopen.bmj.com/site/about/guidelines.xhtml

For peer review only

the usability issues in context and allows designers to address these before potential medical adverse events can occur.

The HSUS is divided into four subscales: patient safety and decision effectiveness, workflow integration, work effectiveness and user control. The free text comments enhance this scale's potential to provide insight into what is in the health professional mindset. As part of the overall analysis, these constructs are useful as it we can now compare systems based on their subscale scores. By determining which construct is the root of the problem, usability concerns may be more easily localized.

ACKNOWLEDGMENTS

We would like to express our sincere acknowledgements to the international panel of experts who evaluated the scale and the usability testing from their homes and institutions in the United States of America (Maryland, Missouri, Pittsburgh, and Utah) and the UK (Manchester, Milton Keynes, and Oxford); Thank you for all the questionnaire respondents as well as the HAVEN research team members who helped in developing and evaluating the HSUS.

COMPETING INTERESTS

- None

DATA SHARING STATEMENT

Data may be available on request from the Chief Investigator

FUNDING

This work was supported by the Health Innovation Challenge Fund grants HICF-R9-524 and WT-103703/Z/14/Z; a parallel funding partnership between the UK Department of Health and

For peer review only - http://bmjopen.bmj.com/site/about/guidelines.xhtml

Social Care and the Wellcome Trust. The views expressed in this publication are those of the authors and not necessarily those of the UK Department of Health and Social Care or the Wellcome Trust.

CONTRIBUTORS

AG was in charge of the study design, data collection, evaluation, analysis and paper writing. JD helped with the study design, paper drafting and proofreading. MW and PW contributed to the study design and approved the final manuscript.

ETHICS APPROVAL

Ethics approval was granted by South Central Oxford C Research Ethics Committee on 13th June 2016 and the REC reference is 16/SC/0264

FIGURE LEGENDS

Figure 1 Phases of development and validation of HSUS scale

Figure 2: Selected examples of improvement suggested by the participants

Figure 3: Background information

Figure 4 Cumulative distribution function of the subscale "Patient safety and decision effectiveness" for SEND system

Figure 5 KMO and Bartelett's Test of the 23 Items scale

Figure 6 Scree plot of the 23 items questionnaire

Figure 7 KMO and Bartlett's Test of the 22 Items scale

For peer review only

References

1. Osheroff JA, et al., *Improving outcomes with clinical decision support: an implementation's guide*. 2nd ed. 2005: Chicago: Healthcare Information and management systems Society (HIMSS).

2. Jaspers, M.W., et al., *Effects of clinical decision-support systems on practitioner performance and patient outcomes: a synthesis of high-quality systematic review findings.* J Am Med Inform Assoc, 2011. **18**(3): p. 327-34.

3. Kaufman, D., et al., *Applying an evaluation framework for health information system design, development, and implementation.* Nurs Res, 2006. **55**(2 Suppl): p. S37-42.

4. Viitanen, J., et al., *National questionnaire study on clinical ICT systems proofs: physicians suffer from poor usability.* International journal of medical informatics, 2011. **80**(10): p. 708-25.

5. Sittig, D.F. and H. Singh, *Defining Health Information Technology-Related Errors New Developments Since To Err Is Human.* Archives of Internal Medicine, 2011. **171**(14): p. 1281-1284.

6. Shibl, R., M. Lawley, and J. Debuse, *Factors influencing decision support system acceptance.* Decision Support Systems, 2013. **54**(2): p. 953-961.

7. Nair, K.M., et al., *A Clinical Decision Support System for Chronic Pain Management in Primary Care: Usability testing and its relevance.* J Innov Health Inform, 2015. **22**(3): p. 329-32.

For peer review only - http://bmjopen.bmj.com/site/about/guidelines.xhtml

8. Kawamoto, K., et al., *Improving clinical practice using clinical decision support systems: a systematic review of trials to identify features critical to success.* BMJ (Clinical research ed), 2005. **330**(7494): p. 765.

9. Trafton, J., et al., *Evaluation of the acceptability and usability of a decision support system to encourage safe and effective use of opioid therapy for chronic, noncancer pain by primary care providers.* Pain Med, 2010. **11**(4): p. 575-85.

10. Genes, N., et al., *Usability Evaluation of a Clinical Decision Support System for Geriatric ED Pain Treatment.* Appl Clin Inform, 2016. **7**(1): p. 128-42.

11. (ISO), I.O.f.S. *ISO/IEC 25010:2011. Systems and software engineering — Systems and software Quality Requirements and Evaluation (SQuaRE) — System and software quality models.* [cited 2019 19 September]; Available from: https://www.iso.org/standard/35733.html.

12. Kushniruk, A.W. and V.L. Patel, *Cognitive and usability engineering methods for the evaluation of clinical information systems.* Journal of biomedical informatics, 2004. **37**(1): p. 56-76.

13. Ghorayeb, A., *ECOVIP: Espace de Communication Visiophonique pour personnes âgées – Conception, Réalisation et Évaluation Participative*, in *Computer Sciences*. 2007, University of Joseph Fourier: Grenoble, France. p. 212.

14. Frøkjær, E., M. Hertzum, and K. Hornbæk. *Measuring usability: are effectiveness, efficiency, and satisfaction really correlated?* in *Proceedings of the SIGCHI conference on Human Factors in Computing Systems*. 2000.

For peer review only

15. Zhang, J., et al., *A cognitive taxonomy of medical errors.* J Biomed Inform, 2004. **37**(3): p. 193-204.

16. Holden, R.J., *Cognitive performance-altering effects of electronic medical records: An application of the human factors paradigm for patient safety.* Cogn Technol Work, 2011. **13**(1): p. 11-29.

17. Tariq, A., et al., *Applying a human factors approach to improve usability of a decision support system in tele-nursing.* Collegian, 2017. **24**(3): p. 227-236.

18. O'Sullivan, D., et al., *Decision time for clinical decision support systems.* Clin Med (Lond), 2014. **14**(4): p. 338-41.

19. Kastner, M., et al., *Usability evaluation of a clinical decision support tool for osteoporosis disease management.* Implement Sci, 2010. **5**: p. 96.

20. Campbell, E.M., et al., *Types of unintended consequences related to computerized provider order entry.* J Am Med Inform Assoc, 2006. **13**(5): p. 547-56.

21. Nebeker, J.R., et al., *High rates of adverse drug events in a highly computerized hospital.* Arch Intern Med, 2005. **165**(10): p. 1111-6.

22. Ratwani, R.M., et al., *Electronic health record usability: analysis of the user-centered design processes of eleven electronic health record vendors.* Journal of the American Medical Informatics Association : JAMIA, 2015. **22**(6): p. 1179-82.

23. McGinn, C.A., et al., *Comparison of user groups' perspectives of barriers and facilitators to implementing electronic health records: a systematic review.* BMC medicine, 2011. **9**: p. 46.

For peer review only

For peer review only - http://bmjopen.bmj.com/site/about/guidelines.xhtml

24. Koppel, R. and D.A. Kreda, *Healthcare IT usability and suitability for clinical needs: challenges of design, workflow, and contractual relations.* Stud Health Technol Inform, 2010. **157**: p. 7-14.

25. Graham, T.A.D., et al., *How usability of a web-based clinical decision support system has the potential to contribute to adverse medical events.* AMIA Annual Symposium proceedings AMIA Symposium, 2008: p. 257-61.

26. Wyatt, J.C. and S.M. Wyatt, *When and how to evaluate health information systems?* International Journal of Medical Informatics, 2003. **69**(2-3): p. 251-259.

27. Yen, P.-Y., *Health information technology usability evaluation: Methods, models, and measures.* 2010, Colombia University: ProQuest Dissertations & Theses Global.

28. Brooke, J., *SUS-A quick and dirty usability scale.* Usability evaluation in industry, 1996. **189**(194): p. 4-7.

29. Horsky, J., et al., *Interface design principles for usable decision support: A targeted review of best practices for clinical prescribing interventions.* Journal of Biomedical Informatics, 2012. **45**(6): p. 1202-1216.

30. Russ, A.L., et al., *A Rapid Usability Evaluation (RUE) Method for Health Information Technology.* AMIA Annu Symp Proc, 2010. **2010**: p. 702-6.

31. Hamborg, K.-C., B. Vehse, and H.-B. Bludau, *Questionnaire based usability evaluation of hospital information systems.* Electronic journal of information systems evaluation, 2004. **7**(1): p. 21-30.

For peer review only - http://bmjopen.bmj.com/site/about/guidelines.xhtml

32. Ellsworth, M.A., et al., *An appraisal of published usability evaluations of electronic health records via systematic review.* Journal of the American Medical Informatics Association, 2017. **24**(1): p. 218-226.

33. Nielsen, J., *Usability engineering*. 1993, Boston ; London: Academic Press. xiv, 358 p.

34. Shneiderman, B., *Designing the user interface : strategies for effective human-computer interaction*. 3rd ed. 1998, Reading, Mass ; Harlow: Addison-Wesley. xiv, 639 p, 12 p of plates.

35. Zhou, L., et al., *The mHealth APP usability questionnaire (MAUQ): development and validation study.* JMIR mHealth and uHealth, 2019. **7**(4): p. e11500.

36. Chin, J.P., V.A. Diehl, and K.L. Norman. *Development of an instrument measuring user satisfaction of the human-computer interface*. 1988. ACM.

37. Meulendijk, M.C., et al., *Computerized Decision Support Improves Medication Review Effectiveness: An Experiment Evaluating the STRIP Assistant's Usability.* Drugs & Aging, 2015. **32**(6): p. 495-503.

38. Knols, B., et al., *The usability aspects of medication-related decision support systems in the inpatient setting: A systematic review.* Health informatics journal, 2019: p. 1460458219841167.

39. Pimentel, M.A., et al., *Detecting deteriorating patients in the hospital: development and validation of a novel scoring system.* American Journal of Respiratory and Critical Care Medicine, 2021. **204**(1): p. 44-52.

For peer review only

For peer review only - http://bmjopen.bmj.com/site/about/guidelines.xhtml

40. Wronikowska, M.W., et al., *Systematic review of applied usability metrics within usability evaluation methods for hospital electronic healthcare record systems: Metrics and Evaluation Methods for eHealth Systems.* Journal of Evaluation in Clinical Practice, 2021. **27**(6): p. 1403-1416.

41. Dahella, S.S., et al., *Implementing a system for the real-time risk assessment of patients considered for intensive care.* BMC Medical Informatics and Decision Making, 2020. **20**(1): p. 1-7.

42. Polit, D.F. and C.T. Beck, *The content validity index: are you sure you know what's being reported? Critique and recommendations.* Research in nursing & health, 2006. **29**(5): p. 489-97.

43. Waltz, C.F., O.L. Strickland, and E.R. Lenz, *Measurement in nursing and health research*. 2010: Springer publishing company.

44. Rubio, D.M., et al., *Objectifying content validity: Conducting a content validity study in social work research.* Social work research, 2003. **27**(2): p. 94-104.

45. Mosier, C.I., *A Critical Examination of the Concepts of Face Validity.* Educational and Psychological Measurement, 1947. **7**(2): p. 191-205.

46. Parsian, N. and T. Dunning, *Developing and validating a questionnaire to measure spirituality: A psychometric process.* Global journal of health science, 2009. **1**(1): p. 2-11.

47. Haladyna, T.M., *Developing and validating multiple-choice test items*. 2004: Routledge.

For peer review only - http://bmjopen.bmj.com/site/about/guidelines.xhtml

48. DeVon, H.A., et al., *A psychometric toolbox for testing validity and reliability.* Journal of Nursing scholarship, 2007. **39**(2): p. 155-164.

49. Lewis, C., *Using the" thinking-aloud" method in cognitive interface design*. 1982: IBM TJ Watson Research Center Yorktown Heights, NY.

50. Darbyshire, J.L., et al., *Monitoring sound levels in the intensive care unit: A mixed-methods system development project to optimize design features for a new electronic interface in the healthcare environment.* International journal of medical informatics, 2021. **153**: p. 104538.

51. McCoach, D.B., R.K. Gable, and J.P. Madura, *Instrument development in the affective domain.* New York, NY: Springer. doi, 2013. **10**: p. 978-1.

52. Bastien, J.M.C., *Usability testing: a review of some methodological and technical aspects of the method.* International journal of medical informatics, 2010. **79**(4): p. e18-23.

53. Yen, P.-Y. and S. Bakken, *Review of health information technology usability study methodologies.* Journal of the American Medical Informatics Association : JAMIA, 2012. **19**(3): p. 413-22.

54. Benedek, J. and T. Miner, *Product reaction cards.* Microsoft, July, 2002. **29**.

55. Bryman, A. and D. Cramer, *Quantitative data analysis with SPSS 14, 15 & 16: A guide for social scientists*. 2009: Routledge/Taylor & Francis Group.

For peer review only

56. Flora, D.B., C. LaBrish, and R.P. Chalmers, *Old and new ideas for data screening and assumption testing for exploratory and confirmatory factor analysis.* Frontiers in Psychology, 2012. **3**: p. 55.

57. Anthoine, E., et al., *Sample size used to validate a scale: a review of publications on newly-developed patient reported outcomes measures.* Health and quality of life outcomes, 2014. **12**(1): p. 1-10.

58. MacCallum, R.C., et al., *Sample size in factor analysis.* Psychological methods, 1999. **4**(1): p. 84.

59. Ros, E.X.R., *FIELD, A.(2005). Discovering Statistics Using SPSS. London: SAGE Publications.* Anuario de psicología/The UB Journal of psychology, 2006. **37**(1): p. 195-196.

60. Truong, Y. and R. McColl, *Intrinsic motivations, self-esteem, and luxury goods consumption.* Journal of Retailing and Consumer Services, 2011. **18**(6): p. 555-561.

61. Bolarinwa, O.A., *Principles and methods of validity and reliability testing of questionnaires used in social and health science researches.* Nigerian Postgraduate Medical Journal, 2015. **22**(4): p. 195.

62. Trochim, W.M. and J.P. Donnelly, *Research methods knowledge base*. Vol. 2. 2001: Atomic Dog Pub.

63. DeVellis, R.F., *Scale development: Theory and applications*. Vol. 26. 2016: Sage publications.

For peer review only - http://bmjopen.bmj.com/site/about/guidelines.xhtml

64. Willis, G.B., *Cognitive interviewing: A tool for improving questionnaire design*. 2004: sage publications.

65. Peterson, C.H., N.A. Peterson, and K.G. Powell, *Cognitive interviewing for item development: Validity evidence based on content and response processes.* Measurement and Evaluation in Counseling and Development, 2017. **50**(4): p. 217-223.

66. Rogers, M.L., et al., *Usability testing and the relation of clinical information systems to patient safety.* 2005.

67. Hertzog, M.A., *Considerations in determining sample size for pilot studies.* Research in nursing & health, 2008. **31**(2): p. 180-191.

68. Julious, S.A., *Sample size of 12 per group rule of thumb for a pilot study.* Pharmaceutical Statistics: The Journal of Applied Statistics in the Pharmaceutical Industry, 2005. **4**(4): p. 287-291.

69. Browne, R.H., *On the use of a pilot sample for sample size determination.* Statistics in medicine, 1995. **14**(17): p. 1933-1940.

For peer review only

For peer review only - http://bmjopen.bmj.com/site/about/guidelines.xhtml

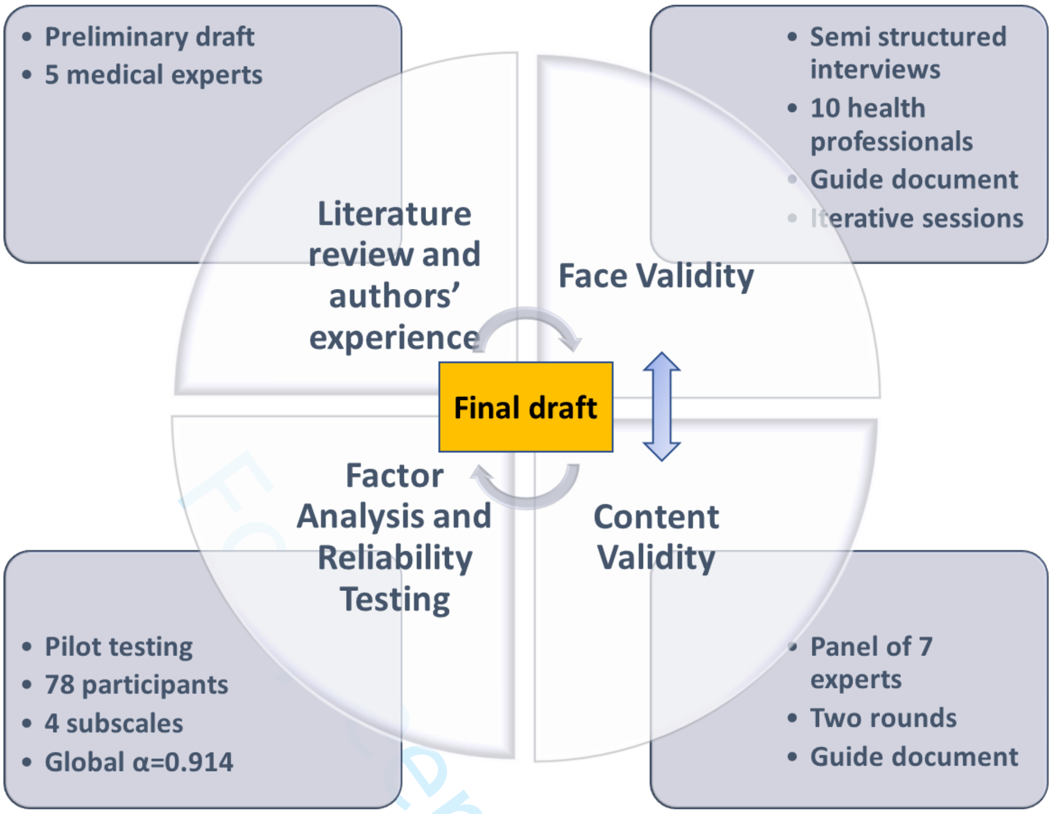

For peer review only

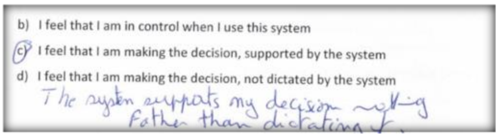
b) I feel that I am in control when I use this system
c) I feel that I am making the decision, supported by the system
d) I feel that I am making the decision, not dictated by the system
The system supports my decision making rather than dictating it.

(a) Suggestion of alternative statements by participant

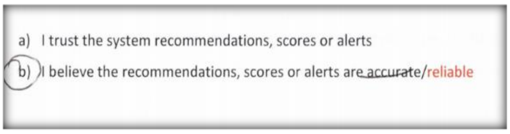
a) I trust the system recommendations, scores or alerts
b) I believe the recommendations, scores or alerts are accurate/reliable

(b) Selection of vocabulary suggested by previous participants

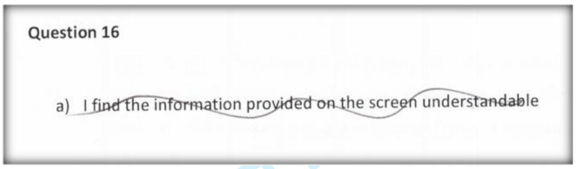
Question 16
a) I find the information provided on the screen understandable

(c) Deletion of repetitive statement

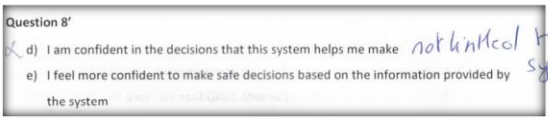
Question 8'
d) I am confident in the decisions that this system helps me make
e) I feel more confident to make safe decisions based on the information provided by the system

(d) Deletion of questions that contradict participants' convictions. Participants confirmed that they are always confident of their decisions.

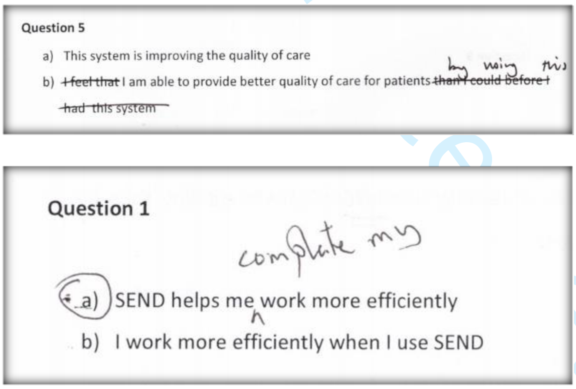
Question 5
a) This system is improving the quality of care
b) I feel that I am able to provide better quality of care for patients than I could before I had this system
Question 1
a) SEND helps me work more efficiently
b) I work more efficiently when I use SEND

(e) Improvement of statements

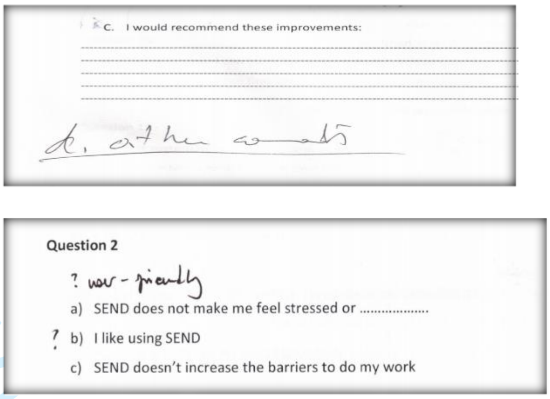
c. I would recommend these improvements:
Question 2
a) SEND does not make me feel stressed or ....
b) I like using SEND
c) SEND doesn't increase the barriers to do my work

(f) Suggestion of a new open-ended question and new statement

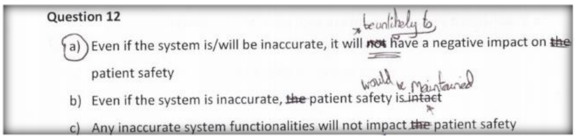
Question 12
a) Even if the system is/will be inaccurate, it will not have a negative impact on the patient safety
b) Even if the system is inaccurate, the patient safety is intact
c) Any inaccurate system functionalities will not impact the patient safety

(g) Suggestion of new vocabularies

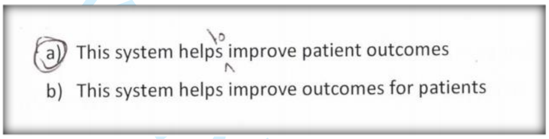
a) This system helps improve patient outcomes
b) This system helps improve outcomes for patients

(h) Spelling/grammar corrections

For peer review only

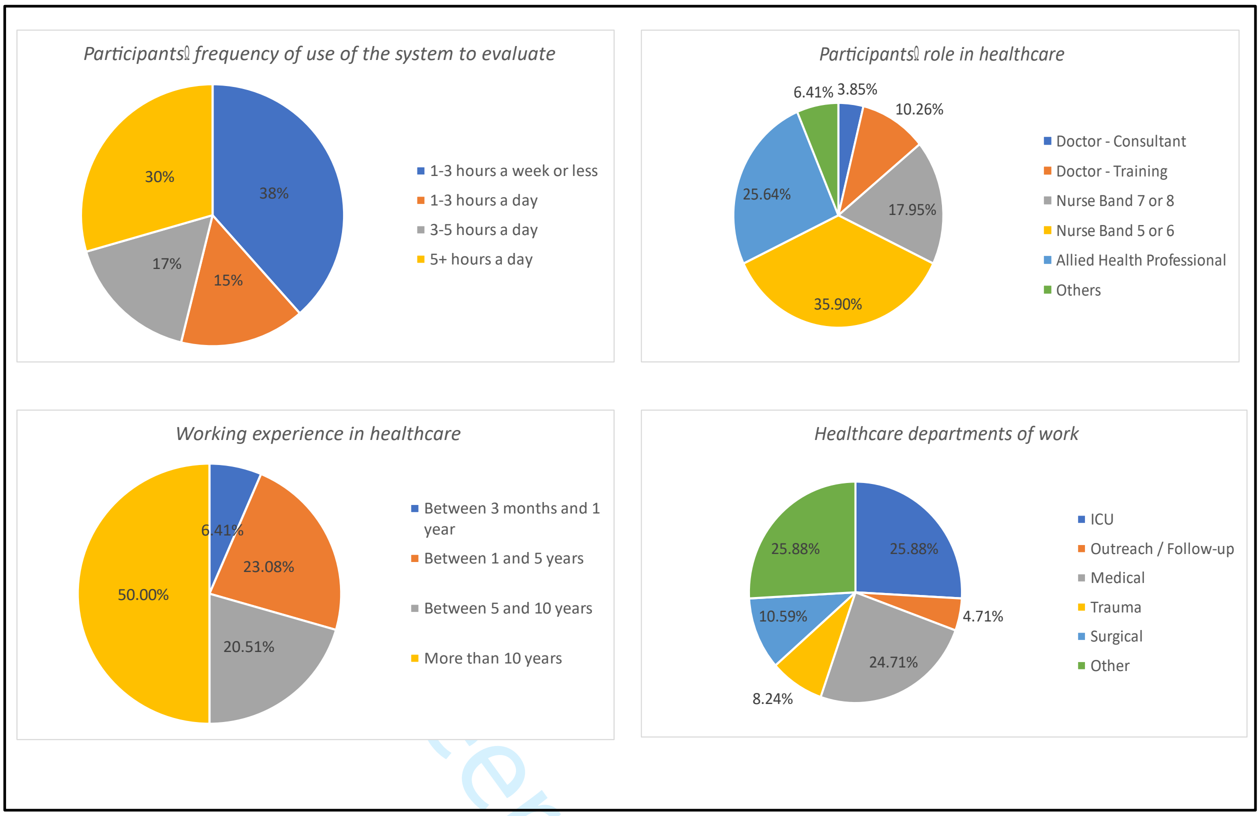

For peer review only

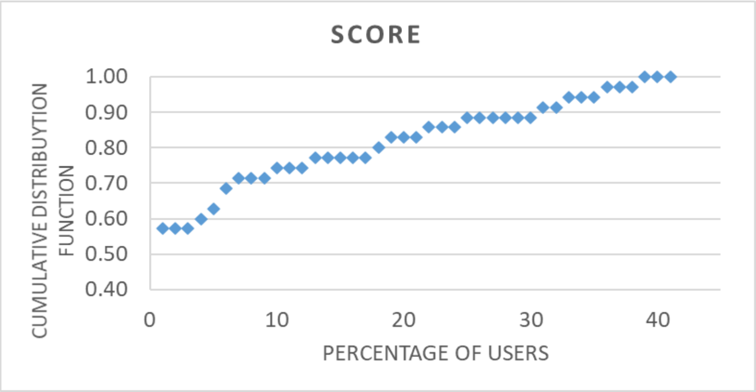

For peer review only

KMO and Bartlett's Test

Kaiser-Meyer-Olkin Measure of Sampling Adequacy		0.818
Bartlett's Test of Sphericity	Approx. Chi-Square	1175.252
	df	253
	Sig.	<0.001

For peer review only

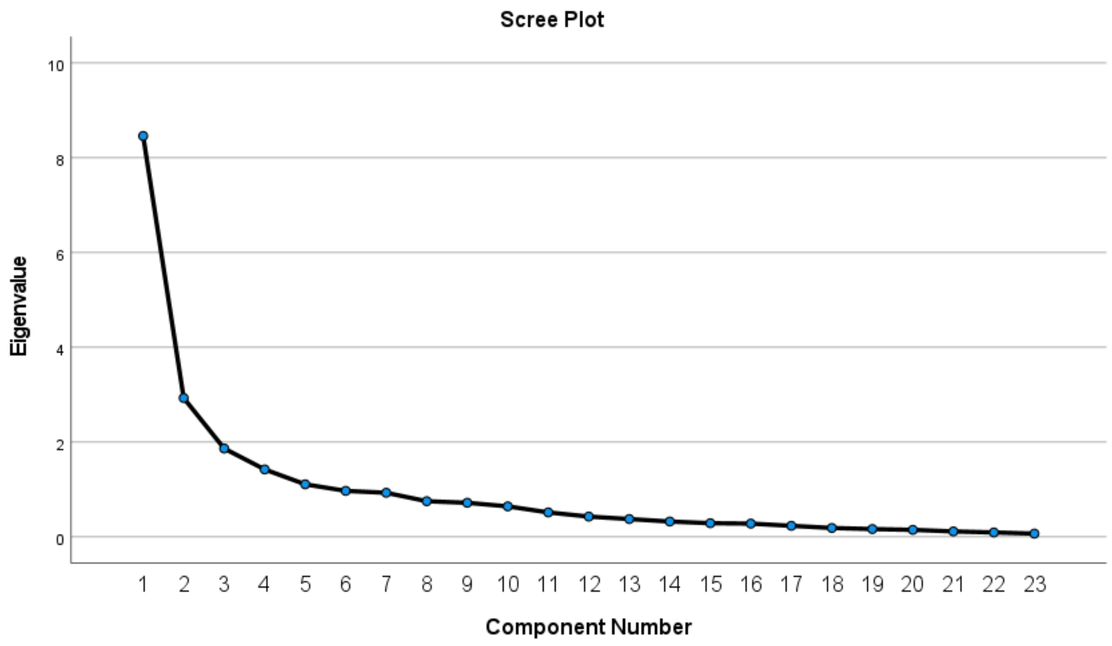

For peer review only

KMO and Bartlett's Test

Kaiser-Meyer-Olkin Measure of Sampling Adequacy		0.823
Bartlett's Test of Sphericity	Approx. Chi-Square	1134.185
	df	231
	Sig.	<0.001

For peer review only

BMJ Open

Design and validation of a new Healthcare Systems Usability Scale (HSUS) for Clinical Decision Support Systems: a mixed methods approach

Journal:	*BMJ Open*
Manuscript ID	bmjopen-2022-065323.R1
Article Type:	Original research
Date Submitted by the Author:	05-Dec-2022
Complete List of Authors:	Ghorayeb, Abir; University of Oxford, Nuffield Department of Clinical Neurosciences<br>Darbyshire, Julie; Oxford University, Department of Primary Care Health Sciences<br>Wronikowska, Marta Weronika; University of Oxford, Nuffield Department of Clinical Neurosciences<br>Watkinson, Peter; Oxford University Hospitals NHS Trust, Kadoorie Centre for Critical Care research and Education
**Primary Subject Heading**:	Health informatics
Secondary Subject Heading:	Health informatics, Patient-centred medicine, Research methods
Keywords:	Information technology < BIOTECHNOLOGY & BIOINFORMATICS, Health informatics < BIOTECHNOLOGY & BIOINFORMATICS, Health & safety < HEALTH SERVICES ADMINISTRATION & MANAGEMENT, QUALITATIVE RESEARCH, Telemedicine < BIOTECHNOLOGY & BIOINFORMATICS, Quality in health care < HEALTH SERVICES ADMINISTRATION & MANAGEMENT

SCHOLARONE™
Manuscripts

I, the Submitting Author has the right to grant and does grant on behalf of all authors of the Work (as defined in the below author licence), an exclusive licence and/or a non-exclusive licence for contributions from authors who are: i) UK Crown employees; ii) where BMJ has agreed a CC-BY licence shall apply, and/or iii) in accordance with the terms applicable for US Federal Government officers or employees acting as part of their official duties; on a worldwide, perpetual, irrevocable, royalty-free basis to BMJ Publishing Group Ltd ("BMJ") its licensees and where the relevant Journal is co-owned by BMJ to the co-owners of the Journal, to publish the Work in this journal and any other BMJ products and to exploit all rights, as set out in our licence.

The Submitting Author accepts and understands that any supply made under these terms is made by BMJ to the Submitting Author unless you are acting as an employee on behalf of your employer or a postgraduate student of an affiliated institution which is paying any applicable article publishing charge ("APC") for Open Access articles. Where the Submitting Author wishes to make the Work available on an Open Access basis (and intends to pay the relevant APC), the terms of reuse of such Open Access shall be governed by a Creative Commons licence – details of these licences and which Creative Commons licence will apply to this Work are set out in our licence referred to above.

Other than as permitted in any relevant BMJ Author's Self Archiving Policies, I confirm this Work has not been accepted for publication elsewhere, is not being considered for publication elsewhere and does not duplicate material already published. I confirm all authors consent to publication of this Work and authorise the granting of this licence.

For peer review only

Design and validation of a new Healthcare Systems Usability Scale (HSUS) for Clinical Decision Support Systems: a mixed methods approach

Abir Ghorayeb*[1], Julie L. Darbyshire[1], Marta Wronikowska[1], Peter J. Watkinson[1, 2]

[1] Critical Care Research Group, Nuffield Department of Clinical Neurosciences, University of Oxford, Oxford, UK.

[2] Adult Intensive Care Unit, Oxford University Hospitals NHS Foundation Trust, Oxford, UK.

**Corresponding Author*

Abir Ghorayeb: Abir.Ghorayeb@bristol.ac.uk

Keywords: Usability scale, SUS, CDSS, mixed research methods, questionnaire, clinical context, digital health

ABSTRACT (300 words)

Objective: To develop and validate a questionnaire to assess the usability of Clinical Decision Support Systems (CDSS) and to assist in the early identification of usability issues that may impact patient safety and quality of care.

Design: Mixed research methods were used to develop and validate the questionnaire. The qualitative study involved scale item development, content, and face validity. Pilot testing

For peer review only - http://bmjopen.bmj.com/site/about/guidelines.xhtml

For peer review only

established construct validity using factor analysis and facilitated estimates for reliability and internal consistency using the Cronbach's alpha coefficient.

Setting: Two hospitals within a single NHS Trust.

Participants: We recruited a panel of 7 experts in usability and questionnaire writing for health purposes to test content validity; 10 participants to assess face validity; and 78 participants for the pilot testing. To be eligible for this last phase, participants needed to be health professionals with at least three months experience using the local hospital electronic patient record system.

Results: Feedback from the face and content validity phases contributed to the development and improvement of scale items. The final HSUS proved quick to complete, easy to understand and was mostly worded by potential users. Exploratory analysis revealed 4 factors related to patient safety, task execution, alerts or recommendations accuracy, the effects of the system on workflow, and ease of system use. These separate into four subscales: patient safety and decision effectiveness (7 items), workflow integration (6 items), work effectiveness (5 items), user control (4 items). These factors affect the quality of care and clinician's ability to make informed and timely decisions when using CDSS. The HSUS has a very good reliability with global Cronbach's alpha 0.914 and between 0.702 and 0.926 for the four subscales.

Conclusion: The HSUS is a valid and reliable tool for usability testing of CDSS and early identification of usability issues that may cause medical adverse events.

STRENGHTS AND LIMITATIONS OF THIS STUDY

- Iterative, user-centred approach, designed with potential users

For peer review only

For peer review only - http://bmjopen.bmj.com/site/about/guidelines.xhtml

- Mixed research methods allow comprehensive assessment of the development and evaluation process
- Panel of experts in usability and questionnaire writing for health purpose review the questionnaire content validity.
- Final scale reliability assessment demonstrates ability of scale to pinpoint usability issues
- Relatively small number of participants for the pilot study

For peer review only

INTRODUCTION (7561 words)

Clinical decision support systems (CDSS) are defined as systems that "*provide clinicians or patients with computer-generated clinical knowledge and patient-related information, intelligently filtered or presented at appropriate times, to enhance patient care*"[1]. To benefit from the extensive capabilities of artificial intelligence, health organisations develop CDSS to improve health outcomes for patients and to assist clinicians make appropriate and informed decisions. Ultimately, the intent of any CDSS is to improve clinical efficiency and safety by avoiding adverse events or errors [2]. Clinicians, often working under pressure, are concerned with relevant information access, efficient task completion and better collaboration or communication with colleagues. The design of the CDSS or health information systems (HIS) in general is critical. Poor usability may significantly impact patient care and safety [3-5]. Usable HIS, and more particularly CDSS, would be expected to reduce medical errors, increase efficiency, reduce the cognitive workload of clinicians, and improve patient safety and care. This can be achieved through intuitive design and easily accessible pertinent information that

For peer review only - http://bmjopen.bmj.com/site/about/guidelines.xhtml

allows users to make timely and informed decisions. Expectations surrounding CDSS development often are too optimistic.

Adoption of CDSS by clinicians is limited due mainly to their poor usability [6, 7], the high volume and complexity of data to analyse, and the failure to fit new systems within existing clinical workflows [6, 8]. Other factors related to system utility, patient safety and impact on professional skills development may also influence the acceptance and use of the CDSS technology [6, 9]. Clinicians need to perceive the benefit of patient safety or improved outcomes to be keen to adopt a new CDSS [9, 10]. They are prepared to learn a hard to use or complex system if it is perceived as useful and helpful in complex situations [6]. However, poor usability may impact patient safety, lead to frustrated users, workflow disruptions, increase of medical errors and decreased efficiency [3-5]. Consequently, designers must be mindful of the various and varying needs of clinicians, taking into consideration patient safety and quality of care.

Usability

Usability is defined by the international organisation for standardisation (ISO) as: "*the extent to which a product can be used by specified users to achieve specified goals with effectiveness, efficiency, and satisfaction in a specified context of use*" [11, 12]. It is generally seen as the ability of the technology to allow users to achieve their tasks safely, effectively, efficiently, and pleasantly within a specified context or environment [13, 14].

Different usability aspects appear to be critical for healthcare system acceptability. Patient safety, prevention of medical errors, information exchange, collaboration between colleagues and the complexity of navigation through the system can all influence the clinician cognitive

For peer review only - http://bmjopen.bmj.com/site/about/guidelines.xhtml

workload. Previous research work on usability of HIS and CDSS suggests that poor usability contributes to the decreased cognitive performance of clinicians [15, 16], low efficiency [17], workflow interruption or disorientation [6, 8, 9, 18, 19], raised medical error risks [20], and higher numbers of adverse events [21]. The electronic health record (EHR), which makes available to clinicians vast amounts of patient data and other information that could be used by CDSS is increasingly suffering from poor usability and safety issues [22, 23]. EHRs are often perceived by users as difficult to use, frustrating, and a source of patient safety hazards, and systems that do not integrate into clinical environments and personal workflow well [24].

Detecting usability issues in the early stages of CDSS development can reduce or highlight human errors and improve patient safety. It is critical to find an adequate way to involve clinicians in the design process from the beginning. However, usability engineering methods are hard to apply and can be time consuming due to the use of various technology systems, complexity of tasks to evaluate, patient and health professionals' recruitment process. They also need to be conducted within a heterogeneous group of healthcare staff with different backgrounds, needs, skills and experiences [4, 25, 26]. Most of the usability evaluation studies of CDSS to date have used conventional usability methods. Interviews or focus groups [27], think-aloud protocols [28], non-validated questionnaires [4, 27], or standardised tools like the Systems Usability Scale (SUS) [28, 29] are all commonly used to evaluate CDSS. To the best of our knowledge, specific standards for evaluating the user centred design of CDSS do not exist [30], and different institutions, organisations or vendors use their own guidelines. For example, Russ et al [31] developed a usability testing method to evaluate health technology software influenced by human factors techniques and a few studies have tried to develop non-validated usability questionnaires to assess the usability of HIS [4, 27, 32]. There is

For peer review only - http://bmjopen.bmj.com/site/about/guidelines.xhtml

therefore a critical need to improve usability standardisation practice for CDSS, and health technology systems in general [22, 33].

Need for a new usability scale for clinical interfaces

Usability is measured by evaluating the interaction between users, tools, tasks, and the context of use. Various researchers have developed measurements tools for usability aspects such as efficiency, satisfaction, and learnability [34, 35]. Questionnaires are the most often applied usability test [36] due to the simplicity of gathering subjective experiences of a large group of participants in a timely and cost-effective way [37]. However, standardised usability questionnaires such as the Questionnaire for User Interface Satisfaction (QUIS) [38] or System Usability Scale (SUS) [29] focus on evaluating the user interface of the system. They do not take into account the wider context of use which is in this case the clinical work [37]. One of the most widely used scale is still the SUS which was originally developed to evaluate the ease of use of systems [29]. The SUS is a standardised questionnaire which consists of ten statements with which participants specify their agreement or disagreement on a Likert scale. Usually, it is used as a primary method of data collection in interface evaluations but sometimes it is one of several methods of data collection.

Due to the lack of a validated tool which takes into consideration the usability aspects related to the clinical context, SUS was the most commonly used scale [36]to assess users' satisfaction of HIS or CDSS [9, 10, 28, 33, 39, 40]. In Nair et al [7] the SUS was applied to evaluate the usability and ease of use of a CDSS for chronic pain management in primary care but identified difficulties in interpretation of the meaning of the score due to the complexity of the clinical task. In the authors' opinion, the SUS neither connects, nor interacts well with the behaviours of the participants observed during the assessment sessions. Another research study [9] used

For peer review only - http://bmjopen.bmj.com/site/about/guidelines.xhtml

an iterative evaluation of a CDSS through two phases in clinical settings and noticed that the system was rated as usable in the first phase and more usable in the second phase on the SUS while the accompanying qualitative study indicates different degrees of usability in each phase. In phase 1 participants reported the interaction with the system was not intuitive, and that it was time consuming to find the pertinent information. Some of them suffered from a lack of information or additional details needed in the decision-making process. Clinical participants considered the systems disruptive to workflow and felt it took them longer to follow the CDSS' recommendations which in turn limited their use of the CDSS. These results were congruent with findings from an earlier phase of the HAVEN project [41] which confirmed that the SUS score may not reflect improvements in interface performance directly. Therefore, the SUS and existent scales are not reflective of the usability in the real world as it does not consider the context of use (in this case, the clinical context). Furthermore, they do not allow evaluators to identify specific usability problems that can be targeted for improvement.

Researchers have previously tried to address this problem by generating their own questionnaire or survey to evaluate the usability of HIS and have used these questionnaires without a clear or systematic validation [4, 27, 32].

A usability scale specifically designed to assess systems in clinical settings can help identify usability problems in an early stage of development or implementation that, if not addressed, may otherwise lead to medical adverse events. To the best of our knowledge, there is no instrument or scale to measure usability of CDSS or HIS that integrates differential usability variance such as patient safety and quality of care. Therefore, there is a critical need for a CDSS/HIS usability scale that takes into consideration different usability aspects, and which is

For peer review only - http://bmjopen.bmj.com/site/about/guidelines.xhtml

also easy and quick to complete and to analyse. An ideal scale would offer an overview of the usability status of the system with some instructions or information highlighting the specific improvement areas on which to focus.

This scale development work is based on a review of the CDSS and HIS usability literature [42] and on our practical experience from usability testing a CDSS system. This Healthcare Systems Usability Scale (HSUS) was designed within the context of the evaluation of a complex CDSS, HAVEN [41, 43]. HAVEN has been designed to be a fully integrated health information system that combines an early warning score with a navigable user interface. Prior to developing the HSUS we evaluated HAVEN for usability, understanding, efficiency, and accuracy. We used the results from these assessments to refine the interface design through an iterative process. Whilst we demonstrated good performance indicators for the HAVEN system, we identified a gap in current evaluation methods. Specifically, we found the common methods used to evaluate usability of information systems lacked specificity in the clinical context. The aim of this paper therefore is to present the processes used to develop and validate a usability scale that takes into consideration the clinical context and is suitable to evaluate the usability of any healthcare system.

METHODS

To develop and validate a new scale there is specific methodology in the literature which combine qualitative and quantitative data effectively [44-50]. These mixed methods have been applied to the development and validation of different scales [50-55]. We used these methods in our study in an exploratory design [56] where we collected qualitative data first and followed this with a quantitative phase. We conclude with a sequential synthesis.

For peer review only - http://bmjopen.bmj.com/site/about/guidelines.xhtml

The HSUS development in this study was iterative. A demographic questionnaire was designed to include a variety of users. To develop a valid, reliable and worthwhile usability scale for HIS in general, and CDSS in particular, mixed research methods were used. The main phases in the development and validation of the HSUS are summarised in Figure 1.

We proceeded as follows:

a) We established the theoretical background based on a literature review and previous usability evaluation sessions of the HAVEN system and authors' experience. As an output of this stage, we developed the first draft of the questionnaire items.

b) We assessed face validity and content validity of the scale using 10 health professionals and a panel of 7 experts in usability and questionnaire writing for health purpose. These were part of the qualitative research study.

c) We analysed the construct validity of the new scale through pilot testing, factor analysis and internal consistency of the scale. This was the basis for the quantitative research study.

Patient and Public Involvement

Two lay members of the public were part of the HAVEN project management committee and were supportive of the need for a Healthcare Systems Usability Scale.

Content validity

A crucial phase in the validity of a new scale is to ensure that key items have not been missed or omitted [57, 58]. The content validity of a questionnaire is usually attained by 'proof reading' by a panel of experts familiar with the construct being measured. Using the panel of

For peer review only - http://bmjopen.bmj.com/site/about/guidelines.xhtml

experts to review the scale items in depth improves its quality, understandability and representativeness. Using six to ten experts is recommended to generate a consensus on the construct of interest [59].

We invited a consulting committee of experts to review the questionnaire content validity. Experts were chosen based on their knowledge of the usability construct and their experience working in healthcare. The panel included experts in usability assessment of HIS, plain language, design of health content, questionnaire design for usability and health purposes, and clinical informatics. An introduction letter and content validation form were created to define the aim of the scale and explain to the experts what we needed from them. The background questionnaire, the Healthcare System Usability Scale, an introductory letter and the content validation form were sent via email. On completion, all items were returned electronically and comments from the expert panel were either integrated into the questionnaire or captured in a separate document. In this phase, we wanted to understand if the questions captured the usability of the CDSS effectively in the clinical context. The experts were asked to simulate filling out the survey while writing notes and comments, check for common errors (such as double-barrelled questions, double negatives, confusing and leading questions), check questions' clearness and relevance to the main construct, check for any missing aspect of the construct in question, check the question distribution and categorisation, and propose any new aspects not covered by the questionnaire. Finally, they were asked to give each question a score from 1 to 10 which refers to the extent each item relates to the usability of the system.

For peer review only - http://bmjopen.bmj.com/site/about/guidelines.xhtml

Face Validity

Face Validity refers to the degree to which the purpose of the scale is obvious to the target population or users and seems to be a valid measure for its purpose [50, 60]. Face validity evaluates the questionnaire in terms of "feasibility, readability, consistency of style and formatting and the clarity of the language used" [61, 62]. Ideally, the participants who assess questionnaires for face validity will be representative of the target population (the end users).

The participants representing future end users who were approached to assess face validity were recruited from two hospitals within the Oxford University Hospitals NHS Foundation Trust (OUHFT), the John Radcliffe Hospital and the Churchill Hospital. We recruited through in-person and email invitation. To be eligible we needed participants to be: (1) Health professionals (physician, nurse, physiotherapist, pharmacist or scientist), 2) Have more than three months experience using the local hospital electronic patient record system (SEND or CERNER Millenium®), (3) Willing to participate in the study.

Prior to the start of the study, all participants signed written informed consent. The question items were presented to allow discussion of the usability and healthcare dimensions to be evaluated or to be included in the scale; ensure the inclusion of any usability missing aspect; review the set of questions and contribute to the usability, understandability and clarity of the question items.

For this project an assessment form was developed to help the interviewer and participants assess questions in terms of clarity, understandability and meaningfulness, layout and style, and the ability of the participants to answer the questions. Face validity of the HSUS was

For peer review only - http://bmjopen.bmj.com/site/about/guidelines.xhtml

determined by a review of the items iteratively by 10 participants. Suggestions from each participant were incorporated in the next iteration evaluated.

Each participant's interview was recorded for analysis. After each interview, the participant's feedback was analysed and the questionnaire was amended to be evaluated by the following participants (by adding, deleting or amending a question item). The original questionnaire consisted of 28 multiple choice questions and four open ended questions. This was tested iteratively with ten health professionals until data saturation was reached.

During the interviews we used the think aloud technique [63]. This requires the participant to explain out loud each thought they have while reading the questions and discussing the different suggested statements. We were looking to learn how participants speak intuitively about system usability, aspects of patient care, and how they interpret statements using their own words. Participants were asked to select the statement they thought best reflected the construct in question, and to change wording, delete statements or add new aspects they thought relevant (Figure 2)**Error! Reference source not found.**.

Pilot testing

The last validation step is to pilot test the scale where participants of the target population complete the questionnaire independently. For the validation and the comparator usability testing of the scale, we used two well-known health systems used at the Oxford University Hospitals NHS Foundation Trust (OUHFT): SEND and CERNER Millenium®. SEND is a CDSS used by health professionals at the hospital to record patient vital signs observations. This is a fully integrated system developed locally. It allows healthcare staff to record and monitor patient

For peer review only - http://bmjopen.bmj.com/site/about/guidelines.xhtml

vital signs electronically from the bedside. The system integrates with the Trust electronic patient record system and incorporates a simple data entry interface with a visual trend of historic values to aid clinical decision making in hospital. The SEND system also includes limited advice to support escalation processes in the event that physiological deterioration is identified. We also asked participants to assess CERNER Millenium® which is a different HIS also in use at the OUHFT. We know from previous evaluations that healthcare staff rate SEND significantly more usable than CERNER Millenium®. Other work has recognised significant limitations in the visual appearance of the CERNER Millenium® interface which is likely to hinder acceptability and satisfaction in comparison to the simplicity of the SEND interface [64].

In the piloted questionnaire, we added the option of “statement not clear” to identify any statement that might need improvement. The option “Not applicable” was also added as not all HIS or CDSS offer the same functionalities. For example, CERNER Millenium® can be used to record and monitor patients’ vital signs observations but does not include a visual graphical display to aid interpretation of data. CERNER Millenium® also includes direct links to the patient admissions and treatment records which SEND does not.

Participants were recruited from two hospitals within the Oxford University Hospitals NHS Foundation Trust (OUHFT), the John Radcliffe Hospital and the Churchill Hospital through in-person and email contacts. The participants represented a range of experience. Eligibility criteria were similar to those recruited to assess the HSUS for face validity and consisted of: (1) Health professionals (physician, nurse, physiotherapist, pharmacist or scientist), 2) Have more than 3 months experience using SEND or CERNER Millenium®, (3) Willing to participate

For peer review only - http://bmjopen.bmj.com/site/about/guidelines.xhtml

in the study. At the beginning of the questionnaire, participants completed and approved a consent form and answered the demographic questions.

The questionnaire was available online and offered as a paper copy. The researchers systematically observed and recorded any difficulties encountered during the process. Data were reviewed to highlight any items that were difficult to understand or generate confusion and to assess the reliability and correlations of the scale. Exploratory factor analyses, which are more convenient for new scales' development [65], were conducted to identify the underlying constructs or factors. Items measuring the same construct were clustered into the same subscale. Finally, the internal consistency and correlation of the whole scale, and for each subscale, was investigated using Cronbach's Alpha coefficient to ensure scale items were consistent.

RESULTS

The first draft of the questionnaire was based on the usability definitions from CDSS literature to cover the different usability aspects including learnability, efficiency, effectiveness, usefulness, accessibility and user satisfaction [66, 67]. We also wanted to acknowledge the clinical context in which this system is intended to be deployed so included additional patient safety and quality of care aspects. The main challenge to creating an effective scale involves writing questions which are unambiguous and understandable using the vocabulary of the target population. This first draft was developed to cover the main usability issues which mainly linked to:

- Understanding how the recommendations, scores or alerts are made [18]: Clinicians emphasise the need to comprehend how the CDSS' decisions, scores or alerts were

For peer review only - http://bmjopen.bmj.com/site/about/guidelines.xhtml

elaborated or developed. It is part of their training to probe for reasons behind results as interpretations of the outcome or critical issues may differ despite apparently identical data. The 'black box' approach is disliked almost universally.

- Ease of access to additional information: The main aim of all CDSS is to provide an intuitive design to allow pertinent information to be easily accessible to facilitate timely informed decisions. Clinicians must be able to locate the specific information they require promptly.

- Perceived utility: one of the main adoption barriers of CDSS is a lack of perception of its utility. Health professionals must feel the new technology will help them improve patient outcomes, improve their decision effectiveness and ensure patient safety.

- Cognitive workload: A CDSS that requires significant mental effort to use is most likely to be rejected by health professionals working in a busy environment.

- Workflow integration: the new system must fit into the workflow of the health professionals and integrate well with current systems and processes. Recommendations, alerts or scores should be provided and displayed in a way that supports the clinical work; otherwise, the system is likely to be seen as a waste of time.

To elaborate the first draft of the questionnaire, we collaborated with five scientists working in the medical field to ensure the inclusion of all construct aspects and the appropriateness of each question.

For peer review only

For peer review only - http://bmjopen.bmj.com/site/about/guidelines.xhtml

Content Validity

The first set of 21 discreet choice questions and four open ended questions was reviewed by the panel of experts. All experts emphasized the need for a new, more clinically focused usability scale to assess CDSS and HIS, as evidenced by the following quotations:

> Expert A: *"I don't know of any survey instrument that focuses specifically electronic decision support systems. Therefore, developing well-researched questionnaires that can help you distinguish between more usable and less usable systems is a worthwhile".*

> Expert B: *"I think the questionnaire can be an excellent contribution to the field. I really like the idea of embedding context as that is critical to perceptions about a system."*

> Expert C: *"I think each of the subscales is relevant to the concept of usability." I also think the respondent burden (the time and effort for respondents to fill out the questionnaire) is fine".*

They commented on the questions' clearness, appropriateness and understandability, and looked for leading, confusing or double-barrelled questions and suggested corrections. The panel of experts were asked to rate each item on a scale of 1 to 10, indicating how closely each item related to the system's usability. We were looking to check whether these items or factors need to be weighted or balanced in some manner. Most experts disagreed with the concept of assigning a score, arguing that it relies on the system being evaluated, its functioning, and role. It is up to the researcher or investigator to decide whether certain needs, such as patient safety and decision effectiveness, should be prioritized. Some items are more important than others and data analysis should take this into account. While the

For peer review only - http://bmjopen.bmj.com/site/about/guidelines.xhtml

HSUS does not weight items, it does allow for the quick and easy identification of the source of usability issues by investigating each subscale separately.

The comments from the expert panel guided us to rethink and reword several of the items. For example, for the first item “I feel satisfied while using the system”, some of the experts suggested to interview potential users with the following: “This question makes me feel …….” and ask users to suggest an adjective or to choose from a range of adjectives using the Microsoft Product Reaction Cards [68]. We were also using the term “complexity of tasks” and the experts wondered how respondents would interpret this. What tasks do they think this system is helping them to manage? Or is the complexity coming from something that’s nothing to do with the system? The participants in the face validity phase proposed we exclude the term “complexity of tasks” and replace this with a daily workload assessment. Other advice included separating legibility, findability and understandability as these are distinct needs.

More generally, the panel of experts guided us through our questionnaire development by suggesting corrections, re-ordering of questions, and advising we needed to be more specific and ask only what is necessary for analysis. They suggested additional questions which might be useful for analysis such experience with electronic healthcare systems other than the specific system being evaluated. They found the open-ended items easy to understand, and thought they would be useful to detect aspects of the construct not otherwise well represented or omitted elsewhere. They also suggested we evaluate the background questionnaire with potential users and ask them for suggestions.

Three of the seven experts offered further review after usability testing of the amended version. This usability testing altered both the content and structure of the questionnaire.

For peer review only - http://bmjopen.bmj.com/site/about/guidelines.xhtml

Subsequently a revised version of the questionnaire was drafted, and a usability and understandability test was developed during the face validity phase. This amended version of the questionnaire was given to the three experts for a second review and a tele-conference was organised to discuss feedback and agree consensus. We made some additional modifications before the pilot testing phase.

Face Validity

The recruited cohort for this phase represented a range of experience, age and skills. The sample consisted of four nurses with different level of expertise, two physiotherapists, two doctors in training, and two consultants. The final scale consists of 22 items rated on a 7-point Likert scale from 'strongly disagree' to 'strongly agree' including 'not applicable' and 'statement not clear'. Data saturation occurred when participants had no more concerns or new suggestions, and we noticed agreement within the last four participants.

We present direct comments and some examples of participants in Figure 2. For example, in question 5, participants were asked to define 'quality of care' to make sure that we share the same meaning. We were asking the participants a series of questions designed to elicit more information about how framing a question in different ways might affect participants' thoughts, understanding and reasons of selection. Each time a person suggested a new item, new terminology, or a statement's correction, the document was changed and reviewed by the next participant. This phase of testing helped us revise some double-barrelled and unclear statements while taking into consideration participants' insights, experience and points of view.

For peer review only - http://bmjopen.bmj.com/site/about/guidelines.xhtml

Participants' responses were summarised on a spreadsheet (Table 1) for more detailed analysis. The last four participants selected the same statement and shared the same understanding. In Table 1 we summarise examples of three statements, selected statements, and justification for the selection.

Table 1 Selected examples of items development during Face validity

User number	Question 2	Question 5	Question 9
01 (senior nurse)	I like using SEND	This system is improving the quality of care	I understand how the system derived its recommendations, scores or alerts
02 (Physiotherapist)	I like using SEND because of its ease of use / because it is stress free	I am able to provide better quality of care for patients than I could before I had this system	I understand how the system derived its recommendations, scores or alerts
03 (pharmacist)	I like using SEND	This system is improving the quality of care	I understand how the system derived its recommendations, scores or alerts
04 (training doctor)	SEND does not make me feel stressed	I feel that I am able to provide better quality of care for patients than I could before I had this system	The system's recommendations are congruent with clinical current practices and standards/ conform
05 (consultant)	SEND does not increase the barriers to do my work	I feel that I am able to provide better quality of care for patients than I could before I had this system	The system's recommendations are consistent with clinical practices and standards
06 (senior nurse)	SEND does not make me feel stressed	My colleagues and I are able to provide better quality of care for patients than we could before we had this system	The system's recommendations are consistent with clinical practices and standards
07 (training doctor)	SEND is user-friendly	I am able to provide better quality of care for patients by using this system	"I understand how the system derived its recommendations, scores or alerts" and "The system's recommendations are consistent with clinical practices and standards". (Clinicians should have been using the system for a while to be able to decide this item)
08 (Doctor)	SEND is user-friendly	I am able to provide better quality of care for patients by using this system	"I understand how the system derived its recommendations, scores or alerts" and "The system's recommendations are consistent with clinical practices and standards".
09 (Scientist)	SEND is user-friendly	This system is improving the quality of care	"I understand how the system derived its recommendations, scores or alerts" and "The system's recommendations are consistent with clinical practices and standards".

For peer review only - http://bmjopen.bmj.com/site/about/guidelines.xhtml

10 (Nurse band 5or 6)	SEND is user-friendly	I am able to provide better quality of care for patients by using this system	"I understand how the system derived its recommendations, scores or alerts" and "The system's recommendations are consistent with clinical practices and standard"
**Selected statement**	**SEND is user-friendly**	**I am able to provide better quality of care for patients by using this system**	**Based on the participants' suggestions. This item is split in two:**<br>**1. I understand how the system derived its recommendations, scores or alerts.**<br>**2. The system's recommendations are consistent with clinical practices and standards**
**Justification**	"I like using SEND": participants might like using the system for many reasons: confusing statement. Participants were hesitating between the two statements: "SEND is easy to use" or "SEND is user friendly". We decided to opt for the last statement as it was suggested by the participants.	The statement, "This system is improving the quality of care", is too broad. Participants can't really know if the system is improving the quality of care, but they can judge their proper work. We think there is no real need to add "I feel" at the beginning of the statement as suggested by two participants.	Pharmacists and physiotherapists were not interested to know about how the scores are derived but they don't mind knowing. Clinicians, by culture, will never use a system if they don't understand a minimum of how the score and recommendations are derived (they said it clearly). Last few participants suggested to keep the original statement and to add the suggested statement as an independent item.

The last version of the amended document was discussed with the three experts who had offered to follow the progress of the work. They advised to proceed with the pilot testing and adopt the suggestions, even though some of these suggestions contradicted the panel of experts' advice. For example, in question 14, many experts suggested to avoid the use of the word "*navigate*" as it is classified as computer jargon and suggested the following statement instead: "*It is easy to get to what I need in this system*". However, during the usability sessions users advised the use of the word "*navigate*" which might be justified by the daily use of computer technology by all the participants and health professionals in general. As a result of the second round of interviews with the panel of experts, a final draft questionnaire was ready for the pilot testing.

For peer review only - http://bmjopen.bmj.com/site/about/guidelines.xhtml

According to the results of the qualitative studies, most of the items were reworded, four items were deleted, and six items were added. The final version of the questionnaire put forward for pilot testing consisted of 23 items.

Pilot testing

The sample consisted of 78 participants aged 18-65 years. Most of the participants use their local electronic healthcare system daily for at least 1-3 hours, have experience in healthcare for more than one year, and work in various healthcare departments as shown in Figure 3.

To investigate if the new HSUS was able to differentiate between a system that is perceived to be hard-to-use (CERNER Millenium®) and a system perceived to be easy-to-use (SEND), we piloted the questionnaire with 37 healthcare professionals using CERNER Millenium® and 41 using SEND. Although the aim was not to evaluate SEND and CERNER Millenium® specifically, the results of the survey revealed that the usability score for CERNER Millenium® was 64 per cent while the general HSUS score for SEND was 81 per cent, with 86 per cent for the workflow integration subscale.

The cumulative distribution function of the subscale "patient safety and decision effectiveness" for SEND system (Figure 4) is an example of the further investigation offered by the HSUS, and it clearly shows a high usability score, with only a few participants scoring less than 80%.

The HSUS included three optional open-ended questions: "*I would add these functions or features*", "*I would change the following*", "*Please use the space below to provide any other comments*". Participants' comments were concise, informative, to the point, and represent the users' perspectives, as demonstrated in Table 2 collected during the pilot testing phase.

For peer review only - http://bmjopen.bmj.com/site/about/guidelines.xhtml

The differing usability scores for SEND and CERNER Millenium® can be understood in context from these user comments.

Table 2 Utility of HSUS open-ended questions: examples from SEND vs CERNER Millenium® cohort

CERNER	SEND
**I needed help with**	
-Navigation, layout, more clinically designed (congruent with systems I am used to)<br>-Is not user friendly-too complex, too many different places to go. For example: tasks/interactive view/care plans- all need to be done every shift for every patient but all in different places and you need to search for them in a big list.<br>-Uploading photos correctly. navigating through the system-finding what relevant to my practice and how to successfully use. it can freeze or be slow. we often miss patients not referred. more information needed upon occasion<br>-Scheduling appointments find notes find coding	-Initial login etc<br>-Nothing<br>-Nothing<br>-Nothing<br>-Nothing<br>-Nothing<br>-Nothing I am happy with using SEND<br>-Nothing currently<br>-Nothing<br>-Correcting data if error used<br>-Nothing
**I would add these functions or features**	
-CERNER should have the "continuous doc" feature added at OUH to make searching much easier<br>-Per shift: all the care plan + tasks + interactive view in the same place and then other things could be added as needed.<br>-instead of having different encounters on the same patient I would have the patient file divided by specialities. Add research function (specialities)	-Total flow whilst on nasal high flow systems<br>-More detailed information regarding normal ranges for individuals. Some 'standard parameters do not apply to all individuals'<br>-Link to labs<br>-Flow rate and FiO2 for high flow nasal oxygen<br>-Accurate recording of patients on HFNC oxygen<br>-Target SpO2 and adjust the traffic light system to reflect this (chronic Respiratory conditions vary so these need to be able to be adjusted.<br>-Flow rate with Airview<br>-Cap refill, Urine output - if applicable<br>-Oxygen therapy should add to the trigger score. Some patients can have a T&T of 0 but be on 8L of oxygen therapy.<br>-If patient has specific O2 ie. COPD aim SATS, to be reflective on SEND screen. Ability to cancel incorrect data after recorded.<br>-When recording blood pressure, there is an option for lying and standing, maybe add the option of sitting up right.<br>-Urine output and fluid balance all under one system to see trends along with the vital signs this may increase compliance.
**I would change the following**	
-Layout, accessibility, observation recording, search function, documentation.<br>-Add on the available functionality such as continuous doc<br>-Not have different encounters -Add a search facility by using coding structure.<br>-Able to build theatre lists and upload Timms data + Bluespier-Add oncology data including chimo radiotherapy	-Better graphic representation<br>-Add in flow rate for high flow oxygen<br>-Increase the login timeout<br>-Make it more like HAVEN<br>-Consideration of link with ICCA for translation of date from one system to the other without duplication. Or the ability to go back further than 4 hrs to input data as it is duplication of records.<br>-Change to NEWs2 regarding oxygen saturations
**Please use the space below to provide any other comments:**	
-It is a poor, pretty unusable system.<br>-more training/update sessions to keep up with the changes. Dr Logon can be temperamental! it slows work efficiently<br>-Generally CERNER has been a fantastic tool to help me to complete my job role. I can quickly check the status of patients and prioritise them accordingly. In order to ensure more staff use the system for patient referrals and communication, it needs to be much more user friendly/replicate the "look/feel" of more established systems. This will ensure patient data is shared safely and securely<br>-When review of medications is requested there is a field to highlight not medically responsible. This generates lots of lists on power note until reviewed by medic but think this should be placed in a different format rather than producing reams of lists<br>-It feels like a very clunky system which is slow and takes a long while to achieve what is required	-Very useful<br>-Nothing to add<br>-I like how if you have starred your patients & discharged them they reappear on your page if readmitted to hospital.<br>-I feel SEND system takes away the clinical thinking and the judgement of staff to make clinical decisions<br>-It takes a bit while to learn and remember about various functions on EPR. Also I find it a bit difficult to correct errors of documentation

For peer review only

-It'is really time consumming, slow, not always making our time efficient seems to be all over the place, needs more structure to the components of nursing care that are needed<br>-The safety messages that pop up when you log in have the "Don't show until new info posted" box but this doesn't work – I keep getting the same message for days or weeks despite using it.	

Factor Analysis

Factor analysis is a statistical method that clusters items into underlying constructs [69]. Each construct is a list of questions or items that can be associated with each other. Authors have argued on exactly how big samples should be as there is no simple rule-of-thumb [70] and it is rarely justified in the literature [71]. Inappropriate sample size might lead to inaccurate findings during the development and validation of a new scale, in particular the identification of the structure of the new scale and its subdivision in latent constructs. The sample can be determined "by the nature of the data, i.e. the stronger the data, the smaller the sample size can be. Strong data display uniformly high communalities without cross-loadings" [72].

To check the sample size's adequacy to undertake exploratory factor analysis for this study, we performed Bartlett's test and the Kaiser-Meyer-Olkin (KMO) measure analyses. The KMO value varies between 0 and 1, with values closer to 0 indicating that factor analysis is inadequate. Exploratory factor analyses, on the other hand, have a high adequacy and can create distinct and reliable subscales or factors when the value is close to one [73]. KMO values between 0.5 and 07 are considered mediocre by Kaiser; 0.7 and 0.8 as good, 0.8 and 0.9 as great, and > 0.9 as superb.

To explore the factor structure of our new HSUS, we used the Principal Component Analysis (PCA) with a Varimax rotation as the extraction method due to its high reliability and lack of error [69]. The inclusion or exclusion of an item in a construct was determined by factor

For peer review only - http://bmjopen.bmj.com/site/about/guidelines.xhtml

loadings >0.5, eigenvalues>1, the scree plot and the total variance are explained by the domain or factor[74].

All 23 items of the HSUS were analysed using exploratory factor analysis. The resulting KMO was 0.818, indicating "great" on the Kaiser scale. The Bartlett test was similarly positive with a p-value <0.001 (Figure 5). As the KMO of the HSUS was above 0.8, and all the variables had loadings >0.5, the sample size is considered adequate for Factor Analysis and can be tested to see whether the scale performs as intended.

When Kaiser's criterion and PCA were applied to the HSUS, five factors had eigenvalues ≥ 1.00. The exploratory factors analysis identified five domains or subconcepts which explained 68.62 % of the data variance. Commonalities of the 23 items ranged from 0.515 to 0.867. Four of the factors had at least three items while the fifth only had one. For a better interpretation we built the scree plot which indicated there were 3 to 5 factors which account for the variance (Figure 6).

Only three statements were judged unclear once by two different participants (*"It is easier to make efficient decisions by using (name of the system); (Name of the system)'s recommendations are consistent with clinical practices and standards; (Name of the system) helps improve patient outcomes".* Factor 5 ("*A recommendation (false alert or score) is unlikely to impact patient safety*") was judged twice as an unclear statement and as this item was covered by the rest of the scale, we decided to delete it to re-assess the statistical analysis. This revised analysis shows the best statistically and conceptually appropriate solution for the new HSUS is a four-factor scale. The KMO of the final 22 item HSUS is 0.823 (Figure 7).

For peer review only - http://bmjopen.bmj.com/site/about/guidelines.xhtml

When Kaiser's criterion and PCA were applied to the 22 items, four factors had eigenvalues ≥1.00 and the EFA results identified 4 factors or sub-concepts where the commonalities of the 22 items ranged from 0.501 to 0.874. This helps to identify and localise the usability issues clearly and easily in each factor.

The first factor evaluates the utility perception of the system. For CDSS this relates to patient safety and decision effectiveness. This factor is formed of seven items, reflecting its importance in system adoption. Factor two includes six items concerned with the ease of use of the system which facilitates integration within the clinical workflow. Factor three with five items reflects the work effectiveness and cognitive workload and the final factor with four items relate to the user's perception of control. The final scale with the factor loadings of the final PCA are shown in Table 3.

For peer review only

Table 3 Final HSUS with factor loadings and Cronbach's Alpha

Items	Utility: Patient safety and decision effectiveness<br>Factor 1	Ease of Use: Workflow Integration<br>Factor 2	Work Effectiveness<br>Factor 3	User Control<br>Factor4
(System name) helps me work more efficiently	0.734			
(System name) makes it easier to collaborate with colleagues	0.758			
I am able to provide better quality of care for patients by using (system name)	0.613			
It is easier to make efficient decisions by using (system name)	0.706			
(System name) helps improve patient outcomes	0.642			
(System name) helps prevent clinical errors	0.677			
(System name) generates a useful summary view of the patient's current health status	0.503			
(System name) fits well with the way I currently work		0.609		
I found the information provided on the screen understandable		0.818		
I found it easy to navigate through (system name)		0.847		
I can easily remember how to use (system name)		0.801		
The screen layout makes it easy to see each piece of information		0.84		
On the screen, I can find specific information I need quickly		0.803		
(System name) helps me prioritise my daily workload			0.675	
I understand how (system name) creates its recommendations (scores or alerts)			0.67	
(System name) 's recommendations (scores or alerts) are consistent with clinical practices and standards			0.704	
(System name) includes all the relevant information I need			0.678	
I believe the recommendations (scores or alerts) are reliable			0.758	
(System name) supports my decision making rather than dictating it				0.516
It is easy to correct a data entry error in (system name)				0.758
(System name) highlights potential data entry errors				0.781
Recommendations (or alerts) do not unnecessarily interrupt my workflow				0.506
**Cronbach's alpha coefficient**	**0.873**	**0.926**	**0.777**	**0.702**

For peer review only

For peer review only - http://bmjopen.bmj.com/site/about/guidelines.xhtml

Internal Consistency and Reliability

To conduct the reliability analysis, we calculated the Cronbach's alpha coefficient which is a measure of the internal consistency of the question scores. It depends on the inter-question correlations and the total number of questions.

Reliability contributes largely to the validity of a questionnaire as it refers to the extent to which the results obtained by the instrument can be reproduced. Internal consistency concerns the degree to which items on the scale or sub-scale are measuring same concept. Reliability can be established using a pilot test with 20 to 30 participants [48]. To study the inter-item correlations within the HSUS we calculated the Cronbach's alpha coefficient [75] which is a measure of the internal consistency of the item scores. Cronbach's alpha was calculated for each subscale as well as for the complete scale. Cronbach's alpha was 0.914 for the HSUS scale, indicating a high inter-items correlation, suggesting the scale is consistently reliable. The Cronbach's alpha of the four subscales also exceeded 0.7, which is the minimum suggested for a new instrument [62, 76] (see Table 3**Error! Reference source not found.**).

DISCUSSION

Challenges in developing a usability scale in the clinical context are significant, and few guidelines exist in the literature. The specificity of the target user is the first challenge to face as health professionals are experiencing growing level of stress and cognitive workload. While new technologies may improve the quality of care and assist health professionals in their decision-making, healthcare professionals will continue to reject any technology that they do not perceive its utility or that does not fit appropriately in their workflow [6, 30]. Another challenge in the healthcare environment is patient safety and quality of care, both of which

For peer review only - http://bmjopen.bmj.com/site/about/guidelines.xhtml

are aspects associated with usability of health information and clinical decision-making systems. When a new healthcare system is established, it should improve patient outcomes while also ensuring the avoidance of errors and adverse effects on patients and/or the wider healthcare system itself. Another challenge is deciding whether these needs must be balanced or whether the necessity of effectiveness (safety, workflow) should be emphasised. This healthcare systems usability scale (HSUS) was specifically created with the intent of taking the context of use into account.

The review of existing CDSS systems, usability scales, and previous research studies urged the need for a short, easy to use, reliable and accurate usability scale for health information systems. To the best of our knowledge, this HSUS is the first validated scale for testing the usability of clinical decision support systems (CDSS) or healthcare information systems (HIS) that also takes into account the specific clinical context. We have shown that the HSUS is an efficient method for collecting subjective opinions and experiences from a large number of potential users, as well as analysing usability issues. Dividing the HSUS into four sub-scales is a novel feature that allows evaluators to identify specific usability issues and investigate each subscale separately. The fact that is easy to fill, brief, and can be delivered electronically makes it simpler to reach large numbers of users. The HSUS was created for health professionals with varying degrees of computer ability or literacy, while keeping in mind that they may be busy, distracted, or interrupted.

This scale can provide critical information to address the most important usability challenges to the design and subsequent use of complex clinical decision support systems. In addition to the HSUS, we recommend that other usability testing methods, such as interviews and focus groups, are also required for a more thorough and realistic evaluation, as well as to evaluate

For peer review only - http://bmjopen.bmj.com/site/about/guidelines.xhtml

whether the participants' behaviours match well with the results from the HSUS. The HSUS will detect early usability issues, identifying the source of problems, and pinpointing the threat rate. It is difficult to assess a system after the initial implementation. Usability scales that are used after a quick review of a new system during a usability test session tend to be less realistic than tools used after a few days or a short-term trial. It may be difficult and inaccurate for users to evaluate systems after their first interaction. It is preferable to use the usability scale a few days after implementation to allow some degree of familiarity to form. This valuable and novel work was developed and validated through several phases

Scale development

The HSUS was developed using a rigorous, iterative, and user-centred approach that consisted of face validity, content validity, exploratory factor analysis, and reliability testing. First, we proposed items based upon our experience from previous evaluation of the HAVEN system. We complimented this knowledge with a literature review and discussions with various clinicians working with the research team. A panel of seven usability experts in forms and surveys, plain language, user experience, and health content designers contributed to the content validity. The number of experts for content validity was sufficient to agree consensus on the main concept [59]. Three of these experts recommended another review of the revised version at the end of the HSUS usability testing or face validity phase.

Face validity of the HSUS was established by an iterative review of the questions by ten health professionals recruited for a semi-structured interview. Each participant's suggestions were incorporated into the following iteration for evaluation, resulting in a total of five iterations of the scale. Face validity was established when we noticed agreement among the last four

For peer review only - http://bmjopen.bmj.com/site/about/guidelines.xhtml

participants. The participant variability and concept saturation determined the sample size for the qualitative work [77, 78].

During the content and face validity phases of the HSUS, items were deleted, changed, and new items were suggested to ensure that all system usability aspects were addressed, and the questionnaire items were well designed and written, with no common errors such as double negative, confusing, or double-barrelled items. The number of items in the HSUS ranged from 21 at the outset to 28 for face validity and finally, after statistical evaluation, to 22. The exploratory factor analysis defined 4 factors: patient safety and decision effectiveness, workflow integration, work effectiveness, user control. These factors covered issues related to the patient safety, the ease of use of the system, task's complexity, the alerts or recommendations utility, the effects of the system on the workflow, the quality of care and ability of the clinician to take an informed and timely decision. The HSUS presented a very good reliability evaluation with a global Cronbach's alpha of 0.914 and subscale alphas ranging from 0.702 and 0.926.

Whilst the open-ended questions were optional, they had several advantages when used within the HSUS questionnaire. They allowed participants to independently and creatively comment using their own lexicon without being influenced by the interviewer and it revealed important issues to investigate further. Free text responses can also provide quick feedback that may reveal unexpected findings.

HSUS subscales

Patient safety and decision effectiveness: CDSS should, in theory, make daily tasks simpler, resulting in increased efficiency and safety. If the performance of health professionals is

For peer review only - http://bmjopen.bmj.com/site/about/guidelines.xhtml

compromised, work efficiency is reduced, and the quality of care and patient safety may be impacted. A lack of usability in this subscale should be concerning, as the usage of this system could compromise patient safety. This part reflects the perceived utility by the health professionals.

Workflow integration: This subscale is more related to the system ease of use while focusing on the health professionals' concerns. In a busy environment, clinicians need a system that fit well in their workflow, understandable and effective in a timely and accurate way.

Work effectiveness: When offering clear, logical, and practical guidance (warning or score) in a way that fit well into the clinical workflow, CDSS have the potential to be extremely useful and improve health professionals work effectiveness.

User control: Health professionals must feel in control of the any new technology system which needs to be responsive to any internal or external change.

Using the HSUS; customization for real world implementation

The online survey was designed to take 10 to 15 minutes to complete due to the time constraints of the health professionals. Some items may not be applicable to any CDSS or HIS. These items can be omitted. The options "unclear statement" and "not applicable" were added for validation purposes. We needed to know if any statements in the HSUS were unclear. For future use of the HSUS, these two options may be omitted, and the final scale will consist of 22 items rated on 5-point Likert scales.

We recommend tailoring the questionnaire to each application and naming the system within the question (replace the word "system"). Depending on the system's functions, the

For peer review only - http://bmjopen.bmj.com/site/about/guidelines.xhtml

questionnaire should be customized to use the terms "recommendations," "scores," or "alerts." (Table 3)

The complementary information gathered from the background questionnaire could be useful for HSUS data analysis. The background questionnaire should be kept short and include only those questions that are directly relevant to the needs of the study.

The HSUS can be used for the assessment of the severity of usability violation of each subscale. Each item is rated on a 1 to 5 scale; 1= Strongly disagree (crucial usability concern), 2= disagree (major usability concern), 3= neutral (minor usability concern), 4= agree (usability can be improved), 5= strongly agree (no usability concerns). A higher score reflects a higher level of agreement on each item. We calculated the overall group average for each item followed by the average for each subscale. The higher the average score for each subscale, the higher the level of usability perceived by participants. The overall score of this scale is the average of the four subscale scores. A usability score between 20% and 50% indicates a critical need to address the system's usability issues; between 50% and 70% indicates a need to address the system's usability concerns, some of which may be major; between 70% and 90% indicates a good usability score with the potential to improve; and between 90% and 100% indicates an excellent and easy to use system. The researcher or investigator must decide if particular requirements, such as patient safety and decision effectiveness, should be prioritized.

Decision effectiveness and patient safety were combined statistically and logically due to the relationship of efficiency to safety [30, 79]. If health professional performance is affected, work efficiency is slowed, and quality of care and patient safety may be jeopardised. A lack of

For peer review only

For peer review only - http://bmjopen.bmj.com/site/about/guidelines.xhtml

usability in this subscale should raise alarms as the use of the system under evaluation might endanger patient safety.

LIMITATIONS

A possible limitation is the relatively small number of participants even though some experts recommend a sample size of 12 to 50 for a pilot study [80-82]. While the KMO-test value revealed that the sample size was sufficient for factor analysis, the findings of the factor analyses may differ with greater number of participants. To strengthen the consistency of the scale and support its standardisation, further work could be undertaken in a larger sample.

This study was limited to healthcare professionals based in the secondary care environment. When used under different contexts or working circumstances, validity testing may be required, but the HSUS could be applied to other types of clinical system in other healthcare settings.

CONCLUSION

In this paper, we have presented novel work to develop a validated and reliable instrument to measure the usability of electronic systems in the healthcare environment. The HSUS can be useful in research institutes and hospitals to improve the usability of a newly designed or implemented CDSS or HIS in an efficient way. When possible, HSUS should be administered a few days following the implementation of the new system to allow for a period of familiarisation. Because it has been designed for self-administration and brevity, this scale offers an easy way to assess the usability of clinical systems that can be used alongside other techniques. This offers a better understanding of the usability issues in context and allows designers to address these before potential medical adverse events can occur.

For peer review only - http://bmjopen.bmj.com/site/about/guidelines.xhtml

For peer review only

The HSUS is divided into four subscales: patient safety and decision effectiveness, workflow integration, work effectiveness and user control. The free text comments enhance this scale's potential to provide insight into what is in the health professional mindset. As part of the overall analysis, these constructs are useful as they allow to compare systems based on their subscale scores. By determining which construct is the root of the problem, usability concerns may be more easily localised.

ACKNOWLEDGMENTS

We would like to express our sincere acknowledgements to the international panel of experts who evaluated the scale and the usability testing from their homes and institutions in the United States of America (Maryland, Missouri, Pittsburgh, and Utah) and the UK (Manchester, Milton Keynes, and Oxford); Thank you for all the questionnaire respondents as well as the HAVEN research team members who helped in developing and evaluating the HSUS.

COMPETING INTERESTS

- None

DATA SHARING STATEMENT

Data may be available on request from the Chief Investigator

FUNDING

This work was supported by the Health Innovation Challenge Fund grants HICF-R9-524 and WT-103703/Z/14/Z; a parallel funding partnership between the UK Department of Health and Social Care and the Wellcome Trust. The views expressed in this publication are those of the

For peer review only - http://bmjopen.bmj.com/site/about/guidelines.xhtml

authors and not necessarily those of the UK Department of Health and Social Care or the Wellcome Trust.

CONTRIBUTORS

AG was in charge of the study design, data collection, evaluation, analysis and paper writing. JD helped with the study design, paper drafting and proofreading. MW and PW contributed to the study design and approved the final manuscript.

ETHICS APPROVAL

Ethics approval was granted by South Central Oxford C Research Ethics Committee on 13th June 2016 and the REC reference is 16/SC/0264

FIGURE LEGENDS

Figure 1 Phases of development and validation of HSUS scale

Figure 2: Selected examples of improvement suggested by the participants

Figure 3: Background information

Figure 4 Cumulative distribution function of the subscale "Patient safety and decision effectiveness" for SEND system

Figure 5 KMO and Bartelett's Test of the 23 Items scale

Figure 6 Scree plot of the 23 items questionnaire

Figure 7 KMO and Bartlett's Test of the 22 Items scale

For peer review only

For peer review only - http://bmjopen.bmj.com/site/about/guidelines.xhtml

References

1. Osheroff JA, et al., *Improving outcomes with clinical decision support: an implementation's guide*. 2nd ed. 2005: Chicago: Healthcare Information and management systems Society (HIMSS).
2. Jaspers, M.W., et al., *Effects of clinical decision-support systems on practitioner performance and patient outcomes: a synthesis of high-quality systematic review findings.* J Am Med Inform Assoc, 2011. **18**(3): p. 327-34.
3. Kaufman, D., et al., *Applying an evaluation framework for health information system design, development, and implementation.* Nurs Res, 2006. **55**(2 Suppl): p. S37-42.
4. Viitanen, J., et al., *National questionnaire study on clinical ICT systems proofs: physicians suffer from poor usability.* International journal of medical informatics, 2011. **80**(10): p. 708-25.
5. Sittig, D.F. and H. Singh, *Defining Health Information Technology-Related Errors New Developments Since To Err Is Human.* Archives of Internal Medicine, 2011. **171**(14): p. 1281-1284.
6. Shibl, R., M. Lawley, and J. Debuse, *Factors influencing decision support system acceptance.* Decision Support Systems, 2013. **54**(2): p. 953-961.
7. Nair, K.M., et al., *A Clinical Decision Support System for Chronic Pain Management in Primary Care: Usability testing and its relevance.* J Innov Health Inform, 2015. **22**(3): p. 329-32.
8. Kawamoto, K., et al., *Improving clinical practice using clinical decision support systems: a systematic review of trials to identify features critical to success.* BMJ (Clinical research ed), 2005. **330**(7494): p. 765.
9. Trafton, J., et al., *Evaluation of the acceptability and usability of a decision support system to encourage safe and effective use of opioid therapy for chronic, noncancer pain by primary care providers.* Pain Med, 2010. **11**(4): p. 575-85.
10. Genes, N., et al., *Usability Evaluation of a Clinical Decision Support System for Geriatric ED Pain Treatment.* Appl Clin Inform, 2016. **7**(1): p. 128-42.
11. (ISO), I.O.f.S. *ISO/IEC 25010:2011. Systems and software engineering — Systems and software Quality Requirements and Evaluation (SQuaRE) — System and software quality models*. [cited 2019 19 September]; Available from: https://www.iso.org/standard/35733.html.
12. Kushniruk, A.W. and V.L. Patel, *Cognitive and usability engineering methods for the evaluation of clinical information systems.* Journal of biomedical informatics, 2004. **37**(1): p. 56-76.
13. Ghorayeb, A., *ECOVIP: Espace de Communication Visiophonique pour personnes âgées – Conception, Réalisation et Évaluation Participative*, in *Computer Sciences*. 2007, University of Joseph Fourier: Grenoble, France. p. 212.
14. Frøkjær, E., M. Hertzum, and K. Hornbæk. *Measuring usability: are effectiveness, efficiency, and satisfaction really correlated?* in *Proceedings of the SIGCHI conference on Human Factors in Computing Systems*. 2000.
15. Zhang, J., et al., *A cognitive taxonomy of medical errors.* J Biomed Inform, 2004. **37**(3): p. 193-204.
16. Holden, R.J., *Cognitive performance-altering effects of electronic medical records: An application of the human factors paradigm for patient safety.* Cogn Technol Work, 2011. **13**(1): p. 11-29.

For peer review only - http://bmjopen.bmj.com/site/about/guidelines.xhtml

17. Tariq, A., et al., *Applying a human factors approach to improve usability of a decision support system in tele-nursing.* Collegian, 2017. **24**(3): p. 227-236.
18. O'Sullivan, D., et al., *Decision time for clinical decision support systems.* Clin Med (Lond), 2014. **14**(4): p. 338-41.
19. Kastner, M., et al., *Usability evaluation of a clinical decision support tool for osteoporosis disease management.* Implement Sci, 2010. **5**: p. 96.
20. Campbell, E.M., et al., *Types of unintended consequences related to computerized provider order entry.* J Am Med Inform Assoc, 2006. **13**(5): p. 547-56.
21. Nebeker, J.R., et al., *High rates of adverse drug events in a highly computerized hospital.* Arch Intern Med, 2005. **165**(10): p. 1111-6.
22. Ratwani, R.M., et al., *Electronic health record usability: analysis of the user-centered design processes of eleven electronic health record vendors.* Journal of the American Medical Informatics Association : JAMIA, 2015. **22**(6): p. 1179-82.
23. McGinn, C.A., et al., *Comparison of user groups' perspectives of barriers and facilitators to implementing electronic health records: a systematic review.* BMC medicine, 2011. **9**: p. 46.
24. Koppel, R. and D.A. Kreda, *Healthcare IT usability and suitability for clinical needs: challenges of design, workflow, and contractual relations.* Stud Health Technol Inform, 2010. **157**: p. 7-14.
25. Graham, T.A.D., et al., *How usability of a web-based clinical decision support system has the potential to contribute to adverse medical events.* AMIA Annual Symposium proceedings AMIA Symposium, 2008: p. 257-61.
26. Wyatt, J.C. and S.M. Wyatt, *When and how to evaluate health information systems?* International Journal of Medical Informatics, 2003. **69**(2-3): p. 251-259.
27. Yen, P.-Y., *Health information technology usability evaluation: Methods, models, and measures.* 2010, Colombia University: ProQuest Dissertations & Theses Global.
28. Poncette, A.-S., et al., *A Remote Patient-Monitoring System for Intensive Care Medicine: Mixed Methods Human-Centered Design and Usability Evaluation.* JMIR Human Factors, 2022. **9**(1): p. e30655.
29. Brooke, J., *SUS-A quick and dirty usability scale.* Usability evaluation in industry, 1996. **189**(194): p. 4-7.
30. Horsky, J., et al., *Interface design principles for usable decision support: A targeted review of best practices for clinical prescribing interventions.* Journal of Biomedical Informatics, 2012. **45**(6): p. 1202-1216.
31. Russ, A.L., et al., *A Rapid Usability Evaluation (RUE) Method for Health Information Technology.* AMIA Annu Symp Proc, 2010. **2010**: p. 702-6.
32. Hamborg, K.-C., B. Vehse, and H.-B. Bludau, *Questionnaire based usability evaluation of hospital information systems.* Electronic journal of information systems evaluation, 2004. **7**(1): p. 21-30.
33. Ellsworth, M.A., et al., *An appraisal of published usability evaluations of electronic health records via systematic review.* Journal of the American Medical Informatics Association, 2017. **24**(1): p. 218-226.
34. Nielsen, J., *Usability engineering*. 1993, Boston ; London: Academic Press. xiv, 358 p.
35. Shneiderman, B., *Designing the user interface : strategies for effective human-computer interaction*. 3rd ed. 1998, Reading, Mass ; Harlow: Addison-Wesley. xiv, 639 p, 12 p of plates.

For peer review only - http://bmjopen.bmj.com/site/about/guidelines.xhtml

36. Hajesmaeel-Gohari, S. and K. Bahaadinbeigy, *The most used questionnaires for evaluating telemedicine services.* BMC medical informatics and decision making, 2021. **21**(1): p. 1-11.
37. Zhou, L., et al., *The mHealth APP usability questionnaire (MAUQ): development and validation study.* JMIR mHealth and uHealth, 2019. **7**(4): p. e11500.
38. Chin, J.P., V.A. Diehl, and K.L. Norman. *Development of an instrument measuring user satisfaction of the human-computer interface*. 1988. ACM.
39. Meulendijk, M.C., et al., *Computerized Decision Support Improves Medication Review Effectiveness: An Experiment Evaluating the STRIP Assistant's Usability.* Drugs & Aging, 2015. **32**(6): p. 495-503.
40. Knols, B., et al., *The usability aspects of medication-related decision support systems in the inpatient setting: A systematic review.* Health informatics journal, 2019: p. 1460458219841167.
41. Pimentel, M.A., et al., *Detecting deteriorating patients in the hospital: development and validation of a novel scoring system.* American Journal of Respiratory and Critical Care Medicine, 2021. **204**(1): p. 44-52.
42. Wronikowska, M.W., et al., *Systematic review of applied usability metrics within usability evaluation methods for hospital electronic healthcare record systems: Metrics and Evaluation Methods for eHealth Systems.* Journal of Evaluation in Clinical Practice, 2021. **27**(6): p. 1403-1416.
43. Dahella, S.S., et al., *Implementing a system for the real-time risk assessment of patients considered for intensive care.* BMC Medical Informatics and Decision Making, 2020. **20**(1): p. 1-7.
44. Yaddanapudi, S. and L. Yaddanapudi, *How to design a questionnaire.* Indian journal of anaesthesia, 2019. **63**(5): p. 335.
45. DeVellis, R.F. and C.T. Thorpe, *Scale development: Theory and applications*. 2021: Sage publications.
46. Boateng, G.O., et al., *Best practices for developing and validating scales for health, social, and behavioral research: a primer.* Frontiers in public health, 2018. **6**: p. 149.
47. Artino, A.R., Jr., et al., *Developing questionnaires for educational research: AMEE Guide No. 87.* Med Teach, 2014. **36**(6): p. 463-74.
48. Bolarinwa, O.A., *Principles and methods of validity and reliability testing of questionnaires used in social and health science researches.* Nigerian Postgraduate Medical Journal, 2015. **22**(4): p. 195.
49. Collingridge, D., *Validating a questionnaire.* Retrieved Febr, 2014. **25**: p. 2017.
50. Parsian, N. and T. Dunning, *Developing and validating a questionnaire to measure spirituality: A psychometric process.* Global journal of health science, 2009. **1**(1): p. 2-11.
51. Tsang, S., C.F. Royse, and A.S. Terkawi, *Guidelines for developing, translating, and validating a questionnaire in perioperative and pain medicine.* Saudi journal of anaesthesia, 2017. **11**(5): p. 80.
52. Bai, Y., et al., *Development and validation of a questionnaire to evaluate the factors influencing training transfer among nursing professionals.* BMC Health Services Research, 2018. **18**(1): p. 1-9.
53. Desalu, O.O., et al., *Development and validation of a questionnaire to assess the doctors and nurses knowledge of acute oxygen therapy.* PLoS One, 2019. **14**(2): p. e0211198.

For peer review only

54. Kaitelidou, D., et al., *Development and validation of measurement tools for user experience evaluation surveys in the public primary healthcare facilities in Greece: a mixed methods study.* BMC family practice, 2019. **20**(1): p. 1-12.
55. Schnall, R., H. Cho, and J. Liu, *Health Information Technology Usability Evaluation Scale (Health-ITUES) for usability assessment of mobile health technology: validation study.* JMIR mHealth and uHealth, 2018. **6**(1): p. e8851.
56. Creswell, J.W., *A concise introduction to mixed methods research*. 2014: SAGE publications.
57. Polit, D.F. and C.T. Beck, *The content validity index: are you sure you know what's being reported? Critique and recommendations.* Research in nursing & health, 2006. **29**(5): p. 489-97.
58. Waltz, C.F., O.L. Strickland, and E.R. Lenz, *Measurement in nursing and health research*. 2010: Springer publishing company.
59. Rubio, D.M., et al., *Objectifying content validity: Conducting a content validity study in social work research.* Social work research, 2003. **27**(2): p. 94-104.
60. Mosier, C.I., *A Critical Examination of the Concepts of Face Validity.* Educational and Psychological Measurement, 1947. **7**(2): p. 191-205.
61. Haladyna, T.M., *Developing and validating multiple-choice test items*. 2004: Routledge.
62. DeVon, H.A., et al., *A psychometric toolbox for testing validity and reliability.* Journal of Nursing scholarship, 2007. **39**(2): p. 155-164.
63. Lewis, C., *Using the" thinking-aloud" method in cognitive interface design*. 1982: IBM TJ Watson Research Center Yorktown Heights, NY.
64. Darbyshire, J.L., et al., *Monitoring sound levels in the intensive care unit: A mixed-methods system development project to optimize design features for a new electronic interface in the healthcare environment.* International journal of medical informatics, 2021. **153**: p. 104538.
65. McCoach, D.B., R.K. Gable, and J.P. Madura, *Instrument development in the affective domain.* New York, NY: Springer. doi, 2013. **10**: p. 978-1.
66. Bastien, J.M.C., *Usability testing: a review of some methodological and technical aspects of the method.* International journal of medical informatics, 2010. **79**(4): p. e18-23.
67. Yen, P.-Y. and S. Bakken, *Review of health information technology usability study methodologies.* Journal of the American Medical Informatics Association : JAMIA, 2012. **19**(3): p. 413-22.
68. Benedek, J. and T. Miner, *Product reaction cards.* Microsoft, July, 2002. **29**.
69. Bryman, A. and D. Cramer, *Quantitative data analysis with SPSS 14, 15 & 16: A guide for social scientists*. 2009: Routledge/Taylor & Francis Group.
70. Flora, D.B., C. LaBrish, and R.P. Chalmers, *Old and new ideas for data screening and assumption testing for exploratory and confirmatory factor analysis.* Frontiers in Psychology, 2012. **3**: p. 55.
71. Anthoine, E., et al., *Sample size used to validate a scale: a review of publications on newly-developed patient reported outcomes measures.* Health and quality of life outcomes, 2014. **12**(1): p. 1-10.
72. MacCallum, R.C., et al., *Sample size in factor analysis.* Psychological methods, 1999. **4**(1): p. 84.

For peer review only

For peer review only - http://bmjopen.bmj.com/site/about/guidelines.xhtml

73. Ros, E.X.R., *FIELD, A.(2005). Discovering Statistics Using SPSS. London: SAGE Publications.* Anuario de psicología/The UB Journal of psychology, 2006. **37**(1): p. 195-196.
74. Truong, Y. and R. McColl, *Intrinsic motivations, self-esteem, and luxury goods consumption.* Journal of Retailing and Consumer Services, 2011. **18**(6): p. 555-561.
75. Trochim, W.M. and J.P. Donnelly, *Research methods knowledge base*. Vol. 2. 2001: Atomic Dog Pub.
76. DeVellis, R.F., *Scale development: Theory and applications*. Vol. 26. 2016: Sage publications.
77. Willis, G.B., *Cognitive interviewing: A tool for improving questionnaire design*. 2004: sage publications.
78. Peterson, C.H., N.A. Peterson, and K.G. Powell, *Cognitive interviewing for item development: Validity evidence based on content and response processes.* Measurement and Evaluation in Counseling and Development, 2017. **50**(4): p. 217-223.
79. Rogers, M.L., et al., *Usability testing and the relation of clinical information systems to patient safety.* 2005.
80. Hertzog, M.A., *Considerations in determining sample size for pilot studies.* Research in nursing & health, 2008. **31**(2): p. 180-191.
81. Julious, S.A., *Sample size of 12 per group rule of thumb for a pilot study.* Pharmaceutical Statistics: The Journal of Applied Statistics in the Pharmaceutical Industry, 2005. **4**(4): p. 287-291.
82. Browne, R.H., *On the use of a pilot sample for sample size determination.* Statistics in medicine, 1995. **14**(17): p. 1933-1940.

For peer review only

For peer review only - http://bmjopen.bmj.com/site/about/guidelines.xhtml

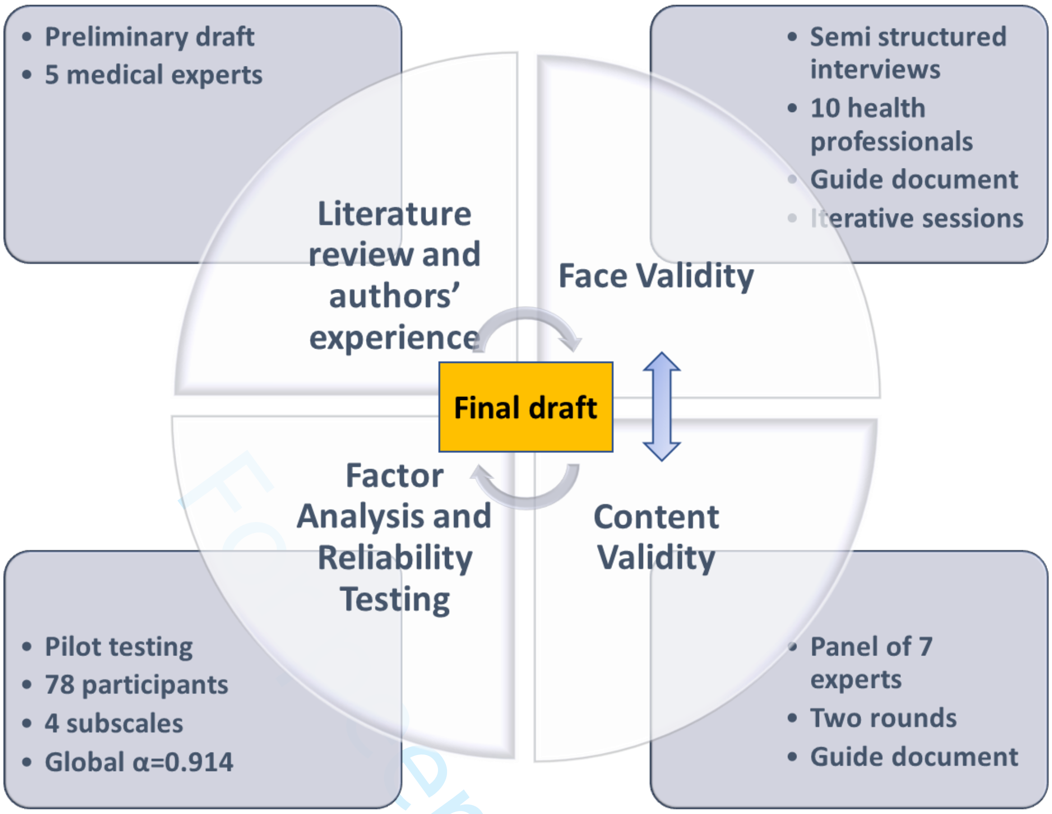

For peer review only

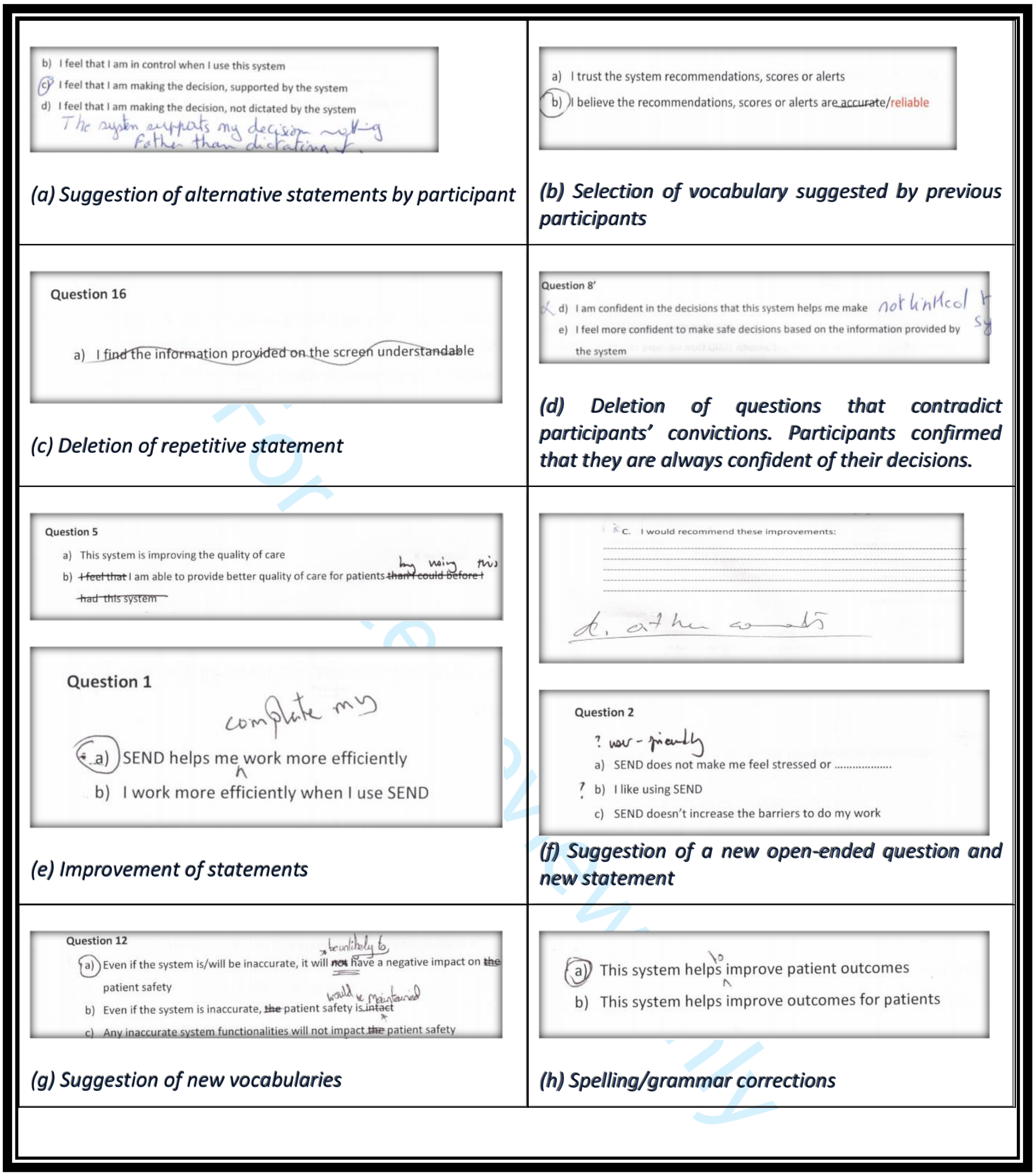

(a) Suggestion of alternative statements by participant

(b) Selection of vocabulary suggested by previous participants

(c) Deletion of repetitive statement

(d) Deletion of questions that contradict participants' convictions. Participants confirmed that they are always confident of their decisions.

(e) Improvement of statements

(f) Suggestion of a new open-ended question and new statement

(g) Suggestion of new vocabularies

(h) Spelling/grammar corrections

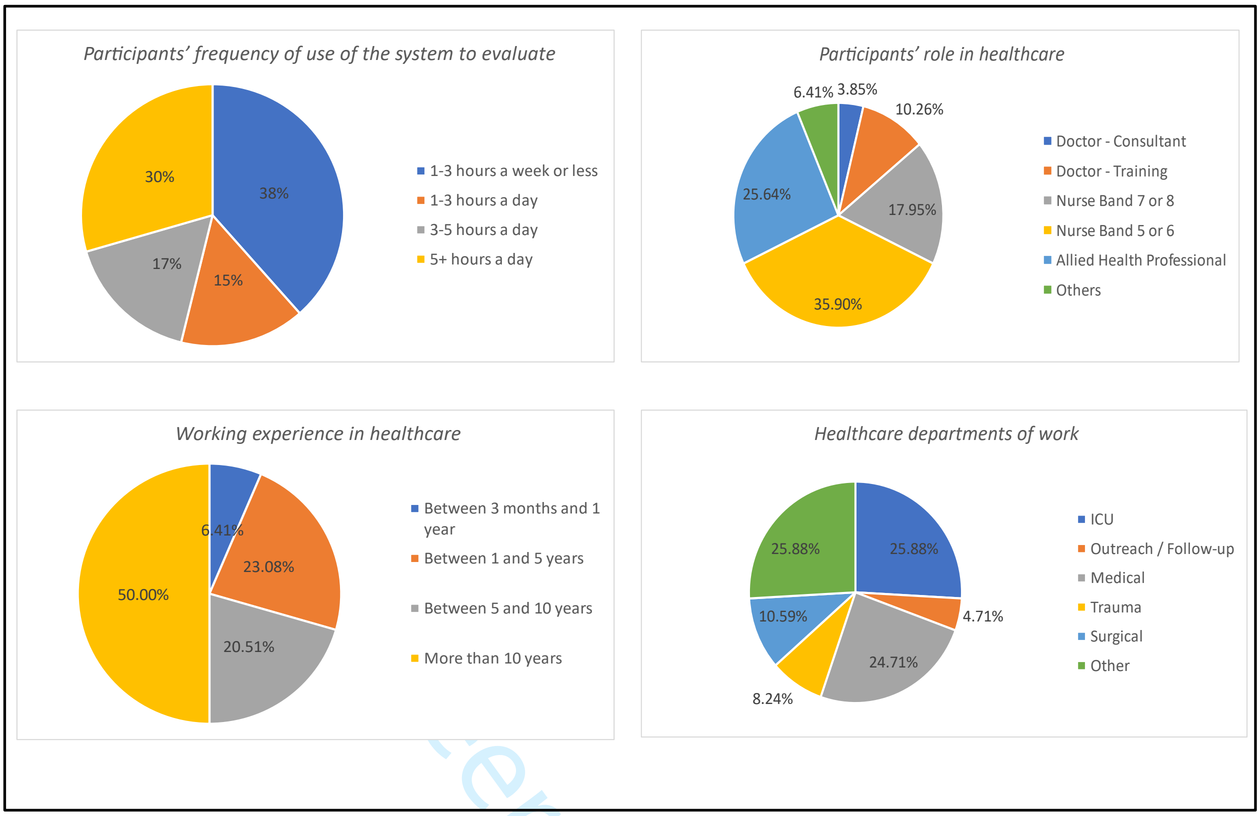
Participants' frequency of use of the system to evaluate
38%
15%
17%
30%
1-3 hours a week or less
1-3 hours a day
3-5 hours a day
5+ hours a day
Participants' role in healthcare
6.41%
3.85%
10.26%
17.95%
35.90%
25.64%
Doctor - Consultant
Doctor - Training
Nurse Band 7 or 8
Nurse Band 5 or 6
Allied Health Professional
Others
Working experience in healthcare
6.41%
23.08%
20.51%
50.00%
Between 3 months and 1 year
Between 1 and 5 years
Between 5 and 10 years
More than 10 years
Healthcare departments of work
25.88%
25.88%
4.71%
24.71%
8.24%
10.59%
ICU
Outreach / Follow-up
Medical
Trauma
Surgical
Other

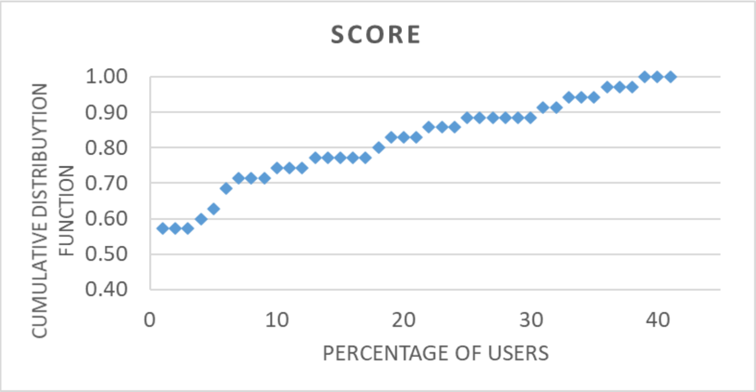

For peer review only

KMO and Bartlett's Test

Kaiser-Meyer-Olkin Measure of Sampling Adequacy		0.818
Bartlett's Test of Sphericity	Approx. Chi-Square	1175.252
	df	253
	Sig.	<0.001

For peer review only

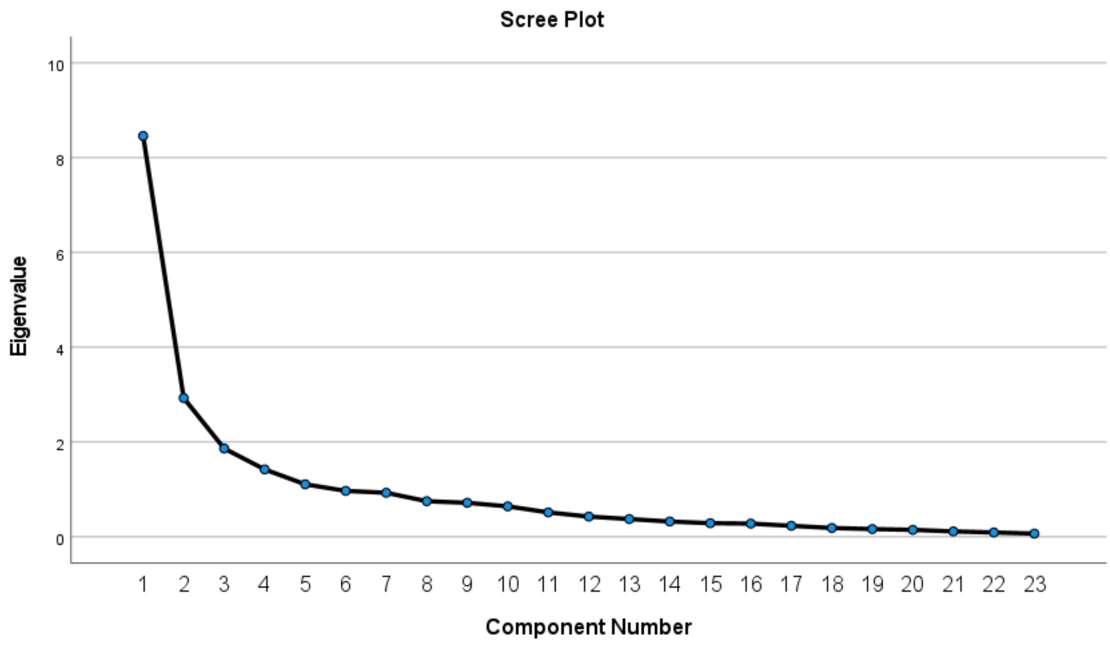

For peer review only

KMO and Bartlett's Test

Kaiser-Meyer-Olkin Measure of Sampling Adequacy		0.823
Bartlett's Test of Sphericity	Approx. Chi-Square	1134.185
	df	231
	Sig.	<0.001

For peer review only
